# Transition metal selenide-based photocatalysts for the degradation of organic dyes: materials design, mechanistic insights, and practical applicability

**DOI:** 10.1039/d6ra04616a

**Published:** 2026-08-14

**Authors:** Nawaz Khan, Waqar Ahmad, Muhammad Khan, Mahnoor Amjid, Adnan Khan, Sabir Khan, Nisar Ali, Nauman Ali, Jiaojiao Zheng

**Affiliations:** a Institute of Chemical Sciences, University of Peshawar Khyber Pakhtunkhwa 25120 Pakistan j.zheng2@aston.ac.uk; b Energy & Bioproducts Research Institute (EBRI), College of Engineering and Physical Sciences (EPS), Aston University Aston Triangle Birmingham B4 7ET UK; c Center of Chemical, Pharmaceutical and Food Sciences, Federal University of Pelotas Pelotas 96010-900 RS Brazil; d Materials Science and Engineering (PPGCEM), Technological Development Center, Federal, University of Pelotas (UFPel) 96010-610 Pelotas RS Brazil; e Department of Basic and Applied Sciences, College of Applied and Health Sciences, A'Sharqiyah University P. O. Box 42 Ibra P. O. 400 Sultanate of Oman

## Abstract

The discharge of organic dyes from industrial effluents poses serious environmental and health risks due to the persistence, toxicity, and poor biodegradability of synthetic dyes, thereby necessitating the development of efficient and sustainable treatment technologies. Photocatalysis has emerged as a green and effective approach for dye degradation under mild operating conditions. In this context, metal selenide-based photocatalysts have gained considerable attention owing to their narrow band gaps, strong visible-light absorption, and efficient charge transport properties. This review provides a comprehensive and critical overview of recent advances in metal selenide-based systems for photocatalytic dye removal. Fundamental aspects of semiconductor photocatalysis are discussed, highlighting light absorption, electron–hole pair generation, and the formation of reactive oxygen species (ROS) such as ˙OH and O_2_˙^−^, which play a central role in oxidative degradation processes. The review systematically examines a wide range of metal selenide materials, including pristine nanostructures, doped systems, heterojunctions, and polymer-supported or hybrid composites, in relation to their photocatalytic performance toward the degradation of various dyes such as methylene blue, rhodamine B, methyl orange, *etc.* Detailed degradation pathways are presented, highlighting key mechanisms such as azo bond cleavage, *N*-dealkylation, hydroxylation, *etc.* Furthermore, key operational parameters influencing photocatalytic efficiency, including pH, catalyst dosage, initial dye concentration, light source, and reaction kinetics, are systematically analysed and correlated with catalyst properties. The impact of material design strategies, including bandgap engineering, surface modification, and heterostructure formation, is critically evaluated to establish structure–property–performance relationships. Finally, the review addresses current challenges related to catalyst stability, recyclability, scalability, and real wastewater applicability, and outlines future perspectives aimed at enhancing solar utilization, durability, and environmental compatibility. Overall, metal selenide-based photocatalysts are identified as highly promising candidates for next-generation, eco-friendly wastewater treatment technologies, providing a strategic pathway toward sustainable environmental remediation.

## Abstract 2

Transition metal selenides are reviewed as visible-light photocatalysts for organic dye degradation, covering material design, mechanisms, kinetics, and practical challenges for sustainable wastewater treatment.

## Graphical abstract

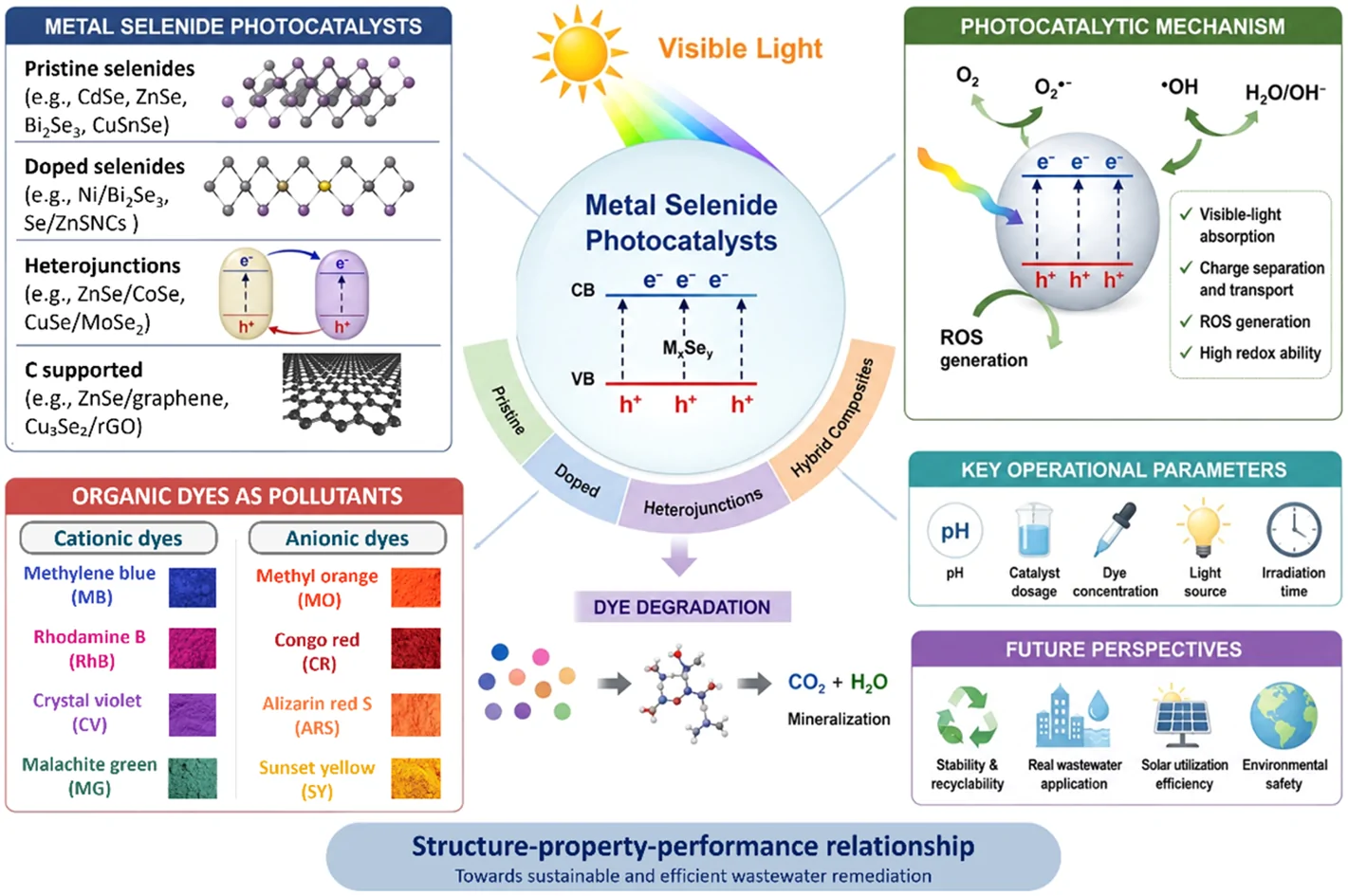

## 1. Introduction

Water, an indispensable resource for life, is increasingly polluted by the indiscriminate disposal of industrial waste into water bodies. Hazardous synthetic dyes and different organic micro-contaminants are found in the wastewater of the food, textiles, and pharmaceutical industries.^1,2^ Thousands of synthetic dyes are continuously released into water bodies, often untreated, causing serious threats to water quality and aquatic ecosystems.^3^ Annually, over one million tons of numerous organic dyes are synthesized worldwide, encompassing over 100 000 dyes available for commercial use.^4^ Annually, the textile industry utilizes 10–12% of these dyes, while 20% is released into wastewater during production and processing.^5,6^ Dye contaminated effluents comprise non-biodegradable, highly toxic colorful pigments that are hazardous to living beings.^7–10^ Dye can cause numerous health problems, such as hypersensitivity, asthma, thyroid damage, hyperactivity in children, illness, sensitivities, and neurotoxicity in both human and animals.^11–13^ The persistence of these dyes not only creates significant challenges for various organisms but also disrupts the balance of aquatic life.^14–16^ Consequently, the effective treatment of wastewater before its release is critical to prevent environmental pollution.^17^ The elimination of dyes and other contaminants from water has grown more difficult in recent times.^7^

Various strategies have been developed to remove dyes from aquatic ecosystems, such as physical methods^18–20^ (adsorption,^21^ filtration^22^ and reverse osmosis,^23^ chemical approaches^24–26^ oxidation,^27^ neutralization,^26^ ion exchange^28^ and biological treatments^29–31^). However, physical and chemical techniques often involve high operational costs and generate toxic sludge, while biological methods face limitations such as slow degradation rates, low efficiency, structural resistance of certain dyes, and microbial tolerance to complex dye molecules.^32,33^ Advanced oxidation processes (AOPs) generate very active oxidizing free radicals, mainly hydroxyl radicals (˙OH). which oxidize organic contaminants into CO_2_ and H_2_O.^34–36^ Photocatalysis is an eco-friendly approach for the elimination of dyes present in industrial wastewater.^37,38^ Recently, semiconductor based photocatalysts have been employed for solar driven applications, enabling efficient degradation of toxic dyes.^39,40^ Photocatalysts enable the effective utilization of solar energy for pollutant degradation, making photocatalytic treatment an economically viable approach.^41,42^

Extensive research has demonstrated the use of a wide range of semiconductor-based materials, including metal oxides, metal sulfides, and ferrites, as photocatalysts for the degradation of organic dyes.^43^ However, their efficiency in achieving complete mineralization of pollutants remains relatively limited.^21^ In this context, transition metal chalcogenides have emerged as a promising class of semiconductors, owing to their versatile physicochemical properties and broad applicability in photovoltaics, photocatalysis, electrocatalysis, optoelectronics, and thermoelectric systems. Among these, selenium-based materials constitute a particularly significant subclass, distinguished by their narrow bandgap and unique electronic structure, which enhance their light-harvesting capabilities.^44^ As an important subgroup of transition metal chalcogenides, metal selenides have recently gained considerable attention due to their superior catalytic performance. Notably, they exhibit remarkable potential in photocatalysis, particularly for the efficient photodegradation of organic pollutants, as well as in electrocatalytic applications.^45,46^ Metal selenide-based photocatalysts offer distinct advantages owing to their narrow tunable band gaps and large surface areas, which provide abundant active sites and enhance quantum efficiency.^47^ The reported band gaps of metal selenides span a remarkably wide range from very narrow values of ∼0.3 eV for Bi_2_Se_3_ and ∼0.9–1.3 eV for SnSe, through ∼1.1–1.5 eV for MoSe_2_ and ∼1.7 eV for CdSe, up to ∼2.7 eV for ZnSe and can be further tuned by controlling composition, crystalline phase, particle size (quantum confinement, *e.g.* ZnSe widening to ∼3.9 eV in nanocrystals), doping, and heterostructure formation.^47^ This breadth allows the absorption onset to be positioned across the near-infrared, visible, and near-UV regions, providing the physical basis for the “tunability” that underpins their visible-light photocatalytic activity. These materials exist in various compositional forms, including binary,^48^ ternary,^49^ and quaternary systems,^50^ depending on the number of constituent metal elements. Among them, quaternary metal selenides have gained particular attention for photocatalytic and electrocatalytic applications due to their enhanced surface characteristics, efficient charge separation, and reduced electron–hole recombination.^47^ Moreover, their strong redox interactions significantly facilitate the conversion of solar energy into chemical energy.^51^

Several published reviews intersect with the subject of this work. Shamraiz *et al.*^4^ surveyed metal sulfides and selenides for dye removal; Sobhani and Salavati-Niasari^45,46^ reviewed the hydrothermal fabrication, morphology, and photocatalytic applications of transition metal selenides and diselenides; Barani *et al.*^22^ reviewed green-synthesized elemental selenium nanoparticles for dye removal, while Roy *et al.*^52,53^ covered both green and conventional syntheses of selenium-based nanomaterials; and, most recently, Manos and Konstantinou^47^ surveyed transition metal selenide catalysts for organic pollutant degradation across advanced oxidation processes more broadly. However, none of these accounts provides a dye-centred, mechanism-oriented analysis devoted specifically to photocatalytic dye degradation over metal selenides. The present review fills this gap in four ways. First, the literature is organized simultaneously by dye class (cationic thiazine, xanthene, and triarylmethane dyes; anionic azo and anthraquinone dyes) and by photocatalyst architecture (binary, ternary, and quaternary selenides; doped systems; carbon, polymer and biopolymer supported composites; and heterojunctions), allowing structure performance trends to be read in both directions. Second, wherever the primary studies permit, performance is compared on a kinetic basis apparent rate constants together with the operating conditions under which they were obtained rather than on percentage degradation alone. Third, dye-specific degradation pathways (azo-bond cleavage, *N*-demethylation/de-ethylation, hydroxylation, and ring opening) are explicitly connected to the reactive oxygen species that selenide systems are thermodynamically able to generate. Fourth, the review critically addresses aspects that are frequently overlooked in this literature: dye-sensitization artefacts in visible-light testing, the distinction between decolorization, degradation, and mineralization, selenium and heavy-metal leaching from spent catalysts, and applicability to real textile effluents. Taken together, these elements make the review useful not only as a survey of results but as a set of design principles for next-generation selenide photocatalysts.

## 2. Recent advances in photocatalyst development for light induced dye degradation

**Fig. 1:**
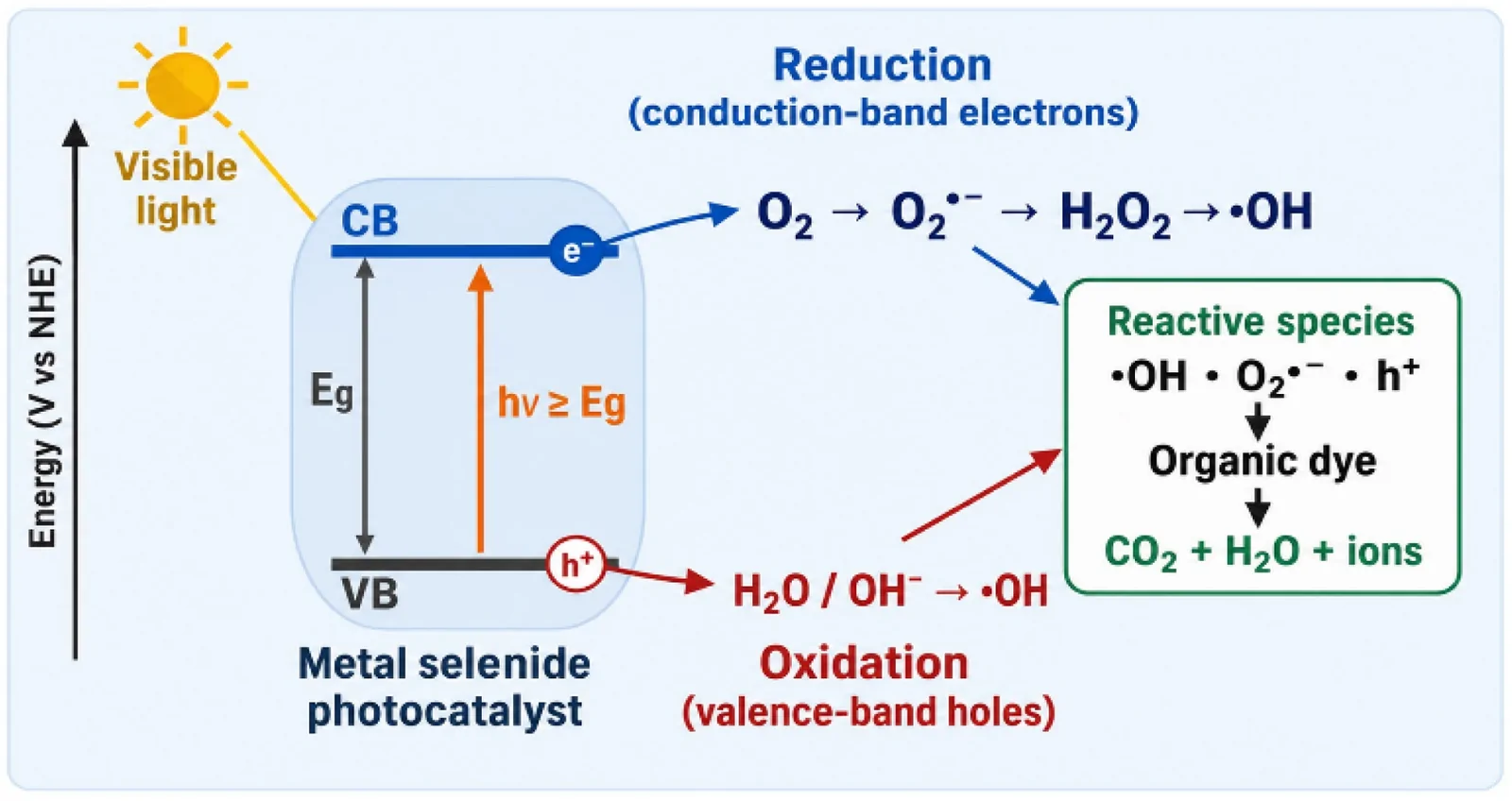
Schematic of the photocatalytic mechanism of a metal selenide semiconductor in aqueous media.

Photochemical processes in aqueous systems are fundamentally governed by light irradiation across the ultraviolet (UV) and visible regions as shown in Fig. 1. Visible-light excitation generates electron–hole pairs that drive reductive (O_2_ → O_2_˙^−^ → H_2_O_2_ → ˙OH) and oxidative (H_2_O/OH^−^ → ˙OH) pathways; the resulting reactive oxygen species mineralize the adsorbed dye to CO_2_, H_2_O, and inorganic ions.

Semiconductor materials can efficiently interact with incident photons when their band gap energies correspond to the energy of the incoming radiation. However, many conventional semiconductors exhibit wide band gaps, confining their light absorption predominantly to the UV region, which constitutes only about 5% of the solar spectrum, thereby limiting their practical applicability. Consequently, beyond possessing an appropriate band gap, an ideal photocatalyst should also exhibit facile synthesis, cost-effectiveness, high photostability, and environmental compatibility.^54^

Nanotechnology offers significant potential for efficient and large-scale wastewater purification due to their high specific surface area, enhanced reactivity, rapid mass transfer, and strong adsorption capacity. Owing to their small size and large surface-to-volume ratio, nanoparticles are particularly well suited for wastewater purification applications. Consequently, nanotechnology has been extensively investigated for its applications in water and wastewater treatment systems.^55^ Over the past few decades, semiconducting nanoparticles have attracted significant research interest due to their distinctive electrical, electronic, catalytic, and optical properties.^56,57^ Semiconductors can act as sensitizers in light-induced redox reactions due to their electronic structure, characterized by a filled valence band and an empty conduction band.^58^ Sunlight-driven photocatalytic degradation of dyes by semiconductor materials signifies one of the most efficient strategies for wastewater remediation.^59^

To date, semiconductor photocatalysts, such as metal oxides, chalcogenides, and other metal salts, as well as their composites, have been extensively developed for dye remediation.^60^ An efficient photocatalyst must have an appropriate band gap to absorb light across a wide spectrum, enable rapid charge transfer for effective redox reactions, and exhibit high stability for repeated use. Furthermore, factors such as permeability, adsorption capacity, surface area, and surface-to-volume ratio critically influence its ability to remove dyes.^61^ The photocatalytic degradation of toxic compounds such as dyes, pesticides, and phenolic substances in aqueous media using photoactive materials mainly depends on factors including band gap energy, surface area, catalyst dosage, and electron–hole pair generation. Among these parameters, surface area plays a dominant role by providing more active sites for dye adsorption on the photocatalyst surface, thereby significantly enhancing photocatalytic activity.^62^

Non-noble transition metal catalysts, including iron,^56^ cobalt,^63^ nickel,^64^ and copper compounds,^65^ have been extensively investigated as promising candidates for AOPs in decontamination technologies. In recent years, transition metal chalcogenides (TMCs), particularly transition metal selenides (TMSe) have garnered significant research interest. TMCs are compounds with the general formula MX, where M is a transition metal and X is a chalcogen elements from Group 16 of the periodic table including oxygen (O), sulfur (S), selenium (Se), tellurium (Te), and polonium (Po).^47^

**Fig. 2:**
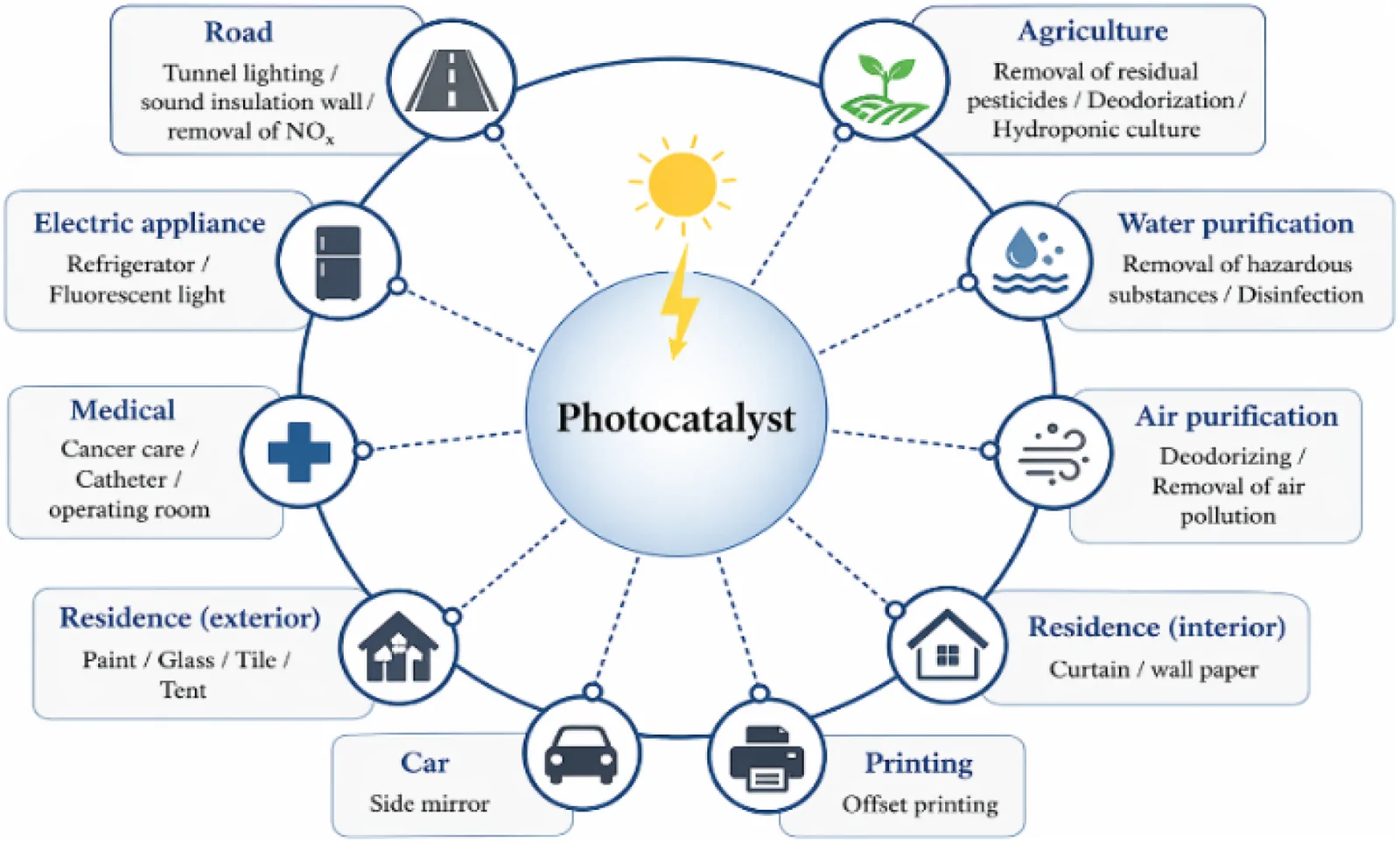
Summary of general applications of semiconductor photocatalysts.^70^

Transition metal selenides offer superior electrical, magnetic, and optical properties, making them promising materials for photocatalytic applications. Various selenides and their composites, including ZnSe,^66^ NiSe_2_–TiO_2_, FeNiSe,^49^ ZnFeSe_2_,^67^ Fe–Bi–Zn–Se^68^ and ZnBiSe,^5^ have been widely explored for photocatalytic and antimicrobial applications. Compared to metal and metal oxide nanoparticles, TMSe possess narrower band gaps, enabling enhanced visible-light absorption, which is essential for efficient photocatalysis.^69^ Photocatalysis is a versatile technology with broad applications, as illustrated in Fig. 2.

## 3. Classification of metal selenide photocatalysts

Beyond grouping by target dye, metal selenide photocatalysts can be classified by their compositional and structural architecture, which offers a more direct route to material-design guidance. On this basis, the systems surveyed here fall into seven broad families binary, ternary, and quaternary selenides; doped selenides; and carbon-, polymer/biopolymer-, and heterojunction-based composites Table 1. Reading the literature along this second axis reveals a consistent trend: performance generally improves as the architecture provides additional means of suppressing electron–hole recombination and extending light absorption, progressing from simple binary phases toward compositionally complex and interfacially engineered systems.

**Table 1:** Classification of metal selenide photocatalysts by compositional and structural architecture, with representative systems and their associated design rationales

Architecture	Representative systems	Design rationale/trend
Binary selenides	CdSe, ZnSe,^71^ SnSe,^72^ NiSe,^44^ CoSe,^73^ TiSe_2_,^74^ Cu_1_._8_Se,^75^ Cu_2−*x*_Se,^76^ Bi_2_Se_3_ (ref. 77)	Baseline activity; narrow gaps but fast e^−^–h^+^ recombination → moderate performance
Ternary selenides	CuSnSe,^76^ Zn_1−*x*_Ni_*x*_Se,^21^ FeNiSe,^78^ Bi–Cu–Se,^79^ Bi–Co–Se,^80^ MnNiSe^45^	Second metal tunes band gap & adds redox couples → better charge separation
Quaternary selenides	AgInSbSe_3_ (ref. 17) quaternary selenide,^81^ Fe–Bi–Zn–Se^68^	Compositional complexity → more active sites, lower recombination
Doped selenides	Ni-doped Bi_2_Se_3_,^82,83^ Ni-doped ZnSe,^84^ Se–ZnSNCs^85^	Dopant states narrow gap/trap carriers → visible-light gains
Carbon-supported	ZnSe/graphene,^86^ PtSe_2_/graphene hybrid materials,^87^ CdSe-rGO nanohybrid,^88^ Cu_3_Se_2_/rGO,^89^ rGO/SnSe^90^	Carbon = electron sink + π–π dye adsorption → suppressed recombination
Polymer/biopolymer-supported	CNF/Ag_2_Se,^91^ CS–PANI,^92^ PANI–Bi_2_Se_3_,^93^ chitosan microspheres.^5^ Chitosan–ZnSe^66^	Support gives adsorption, dispersion, recyclability, easy recovery
Heterojunctions	ZnSe/CoSe,^94^ CuSe/MoSe_2_,^95^ MoSe_2_/ZnSe,^96^ ZnO/ZnSe/MoSe_2_,^88^ Fe_3_O_4_/ZnO/ZnSe^97^	Band alignment drives directional charge transfer → highest performances

### 3.1 Architectural classes

Binary selenides (MSe) such as CdSe, ZnSe, SnSe, NiSe, CoSe, TiSe_2_, and Bi_2_Se_3_ form the baseline of the field. Their narrow band gaps enable visible-light harvesting, but rapid charge-carrier recombination and, in several cases, photocorrosion limits their standalone efficiency.^44,71–74,77^ They are therefore most useful as the active phase that the remaining architectures seek to improve.

Ternary selenides introduce a second metal that tunes the band gap and adds redox-active couples promoting charge separation. Systems such as Zn_1−*x*_Ni_*x*_Se,^21^ and chitosan-supported Fe–Ni, Fe–Bi, and Bi–Cu selenides.^78–80^ Consistently outperform their binary counterparts, several reaching ∼99% dye removal under sunlight,^5,78^ Quaternary selenides (*e.g.* AgInSbSe_3_,^17^ Fe–Bi–Zn–Se).^68^ Extend this logic; their compositional complexity multiplies active sites and lowers recombination, although synthesis is harder to control and structure–activity relationships remain poorly defined.

Doped selenides use a foreign element to introduce mid-gap states or trap carriers. Ni-doping of Bi_2_Se_3_ is the clearest case, enhancing visible-light activity so sharply that complete malachite-green degradation was achieved within 5 min (*k* = 1.21 min^−1^), far exceeding the pristine phase.^83^

Carbon-supported composites exploit graphene/rGO as an electron sink and a π–π adsorption platform. Incorporating rGO raised SnSe performance from 83.5% to 95.5%,^90^ with analogous gains for CdSe/rGO and Cu_3_Se_2_/rGO hybrids,^89,98^ confirming carbon mediation as a reliable route to suppressed recombination.

Polymer- and biopolymer-supported systems polyaniline composites.^93,99^ and, most commonly, chitosan and cellulose microspheres.^5,78,91^ Contribute dye adsorption, catalyst dispersion, and, crucially, mechanical recyclability, addressing the catalyst-recovery problem that limits powder photocatalysts.

Heterojunction photocatalysts are the best-performing family. Coupling two semiconductors with matched band edges (*e.g.* ZnSe/CoSe,^94^ CuSe/MoSe_2_,^95^ MoSe_2_/ZnSe,^96^ ZnO/ZnSe/MoSe_2_ (ref. 88)) drives directional interfacial charge transfer; ZnSe/CoSe reached 99.4% degradation in 120 min, and CuSe/MoSe_2_ gave a 4.5-fold enhancement over pristine MoSe_2_. The specific charge-transfer pathways underpinning these systems are examined in Section 7.

Taken together, the literature reveals a clear hierarchy: pristine binary selenides provide modest baseline activity, whereas compositional complexity (ternary/quaternary/doping) and, above all, interfacial engineering (carbon mediation and heterojunction formation) deliver the largest and most reproducible gains a trend that directly informs the rational design of next-generation selenide photocatalysts.

### 3.2 Related systems: elemental selenium and selenium-doped materials

Several of the studies discussed in this review employ elemental selenium (Se^0^) nanostructures or selenium-containing materials that are not, strictly speaking, transition metal selenides. Although outside the primary scope defined by the review's title, they are included here for context and comparison, as they share the same selenium-based redox chemistry and target the same organic dyes. Velayati *et al.*^122^ synthesized uniform Se nanorods *via* a green approach using gum Arabic and ascorbic acid, achieving 85% RhB degradation under UV-A irradiation. Cytotoxicity studies confirmed their safety up to 1400 µg mL^−1^, suggesting potential biomedical applications. In a related study, Velayati *et al.*^123^ developed chitosan-stabilized Se nanorods through an eco-friendly synthesis route, achieving similar degradation efficiency (85%) while demonstrating favorable biocompatibility, with IC_50_ values of 750 and 500 µg mL^−1^ for CT-26 and HT-29 cell lines, respectively. Che *et al.*^124^ reported the biogenic synthesis of selenium nanomaterials using Lysinibacillus sp. ZYM-1. Monoclinic Se nanosheets exhibited superior visible-light-driven RhB degradation (*k* = 0.0048 min^−1^), along with a photomemory effect and dual degradation pathways involving *N*-deethylation and chromophore cleavage, emphasizing their potential as eco-friendly photocatalysts for wastewater treatment. Similarly, Anitha *et al.*^120^ developed ZnSeO_3_ catalysts using plant extracts (*Nephrolepis cordifolia* and *Ziziphus jujuba*), demonstrating up to 99% degradation of RhB and oxytetracycline, along with strong antibacterial activity. This enhanced performance was primarily associated with improved surface reactivity and effective charge carrier separation, underscoring the potential of green-synthesized materials for sustainable wastewater remediation. Saied *et al.*^102^ synthesized selenium nanoparticles (SeNPs) using a cell-free extract of Aspergillus terreus through an eco-friendly route. The biogenic SeNPs exhibited notable antimicrobial activity against both Gram-positive and Gram-negative bacteria, as well as fungi (inhibition zones of 12–20 mm), while achieving 89% photocatalytic degradation of malachite green. These findings highlight the dual functionality of selenium-based nanomaterials in environmental remediation and antimicrobial applications. In a complementary green synthesis approach, Hassanien *et al.*^103^ biosynthesized selenium nanoparticles (Se-NPLs) using Moringa oleifera leaf extract. The biogenic nanoparticles exhibited 83.8% degradation of sunset yellow under solar irradiation, alongside notable anticancer activity against Caco-2, HepG2, and MCF-7 cell lines. These findings highlight the dual functionality of selenium-based nanomaterials in environmental remediation and biomedical applications. In another study, Ahluwalia *et al.*^85^ synthesized Se–ZnS nanocomposites *via* wet impregnation followed by calcination. The incorporation of 1 wt% Se increased the surface area (from 54 to 75 m^2^ g^−1^) and induced a red-shift in the bandgap (3.77 to 3.30 eV), significantly enhancing photocatalytic performance. The composite achieved 95% degradation of methyl orange, markedly outperforming pristine ZnS (55%), and followed pseudo-first-order kinetics (*k* = 8.42 × 10^−3^ min^−1^), underscoring the effectiveness of selenium incorporation in improving photocatalytic efficiency.

## 4. Synthesis, characterization, and structure–property relationships

The synthesis route, resulting microstructure, and physicochemical properties of metal selenides are inseparable from their photocatalytic behaviour. This section compares the principal preparation methods, outlines the characterization toolkit used to establish structure, and draws out the structure–property relationships that convert the empirical performance trends of later sections into transferable design rules.

### 4.1 Synthesis methods

**Fig. 3:**
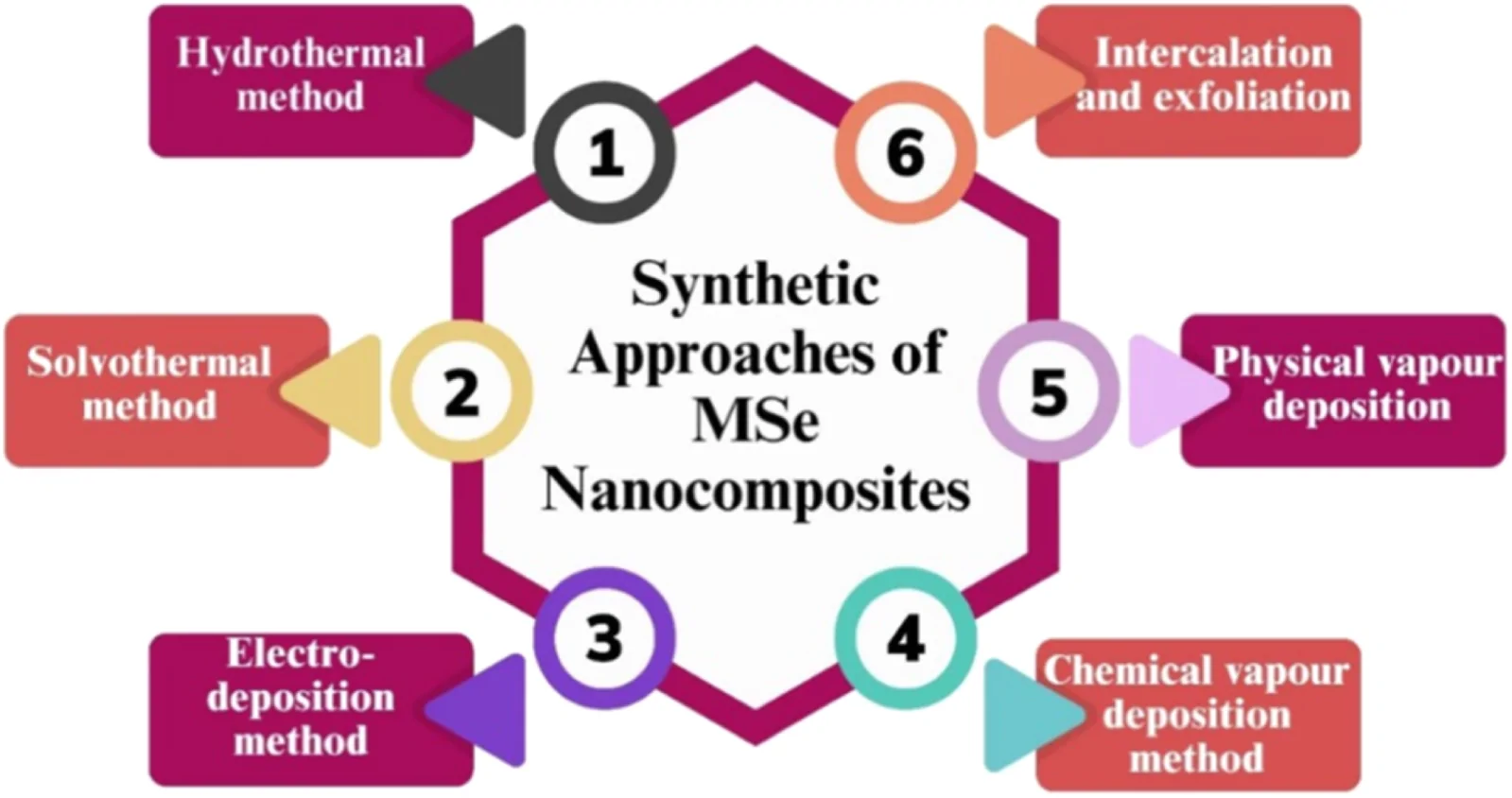
Principal synthesis approaches for metal selenide nanocomposites: hydrothermal, solvothermal, electrodeposition, chemical and physical vapour deposition, and intercalation or exfoliation. Reproduced with permission from ref. 100. Copyright 2025, Elsevier.

Metal selenides have been prepared by a broad range of routes, each imparting characteristic morphology, crystallinity, and defect structure that govern photocatalytic behaviour Table 2. Various approaches used for synthesis are summarised in Fig. 3.

**Table 2:** Comparison of synthesis methods for metal selenide photocatalysts and their influence on product characteristics

Method	Effect on product	Advantages	Limitations	Examples
Co-precipitation	Low crystallinity, agglomeration; needs annealing	Simple, scalable, cheap	Poor size control	71 and 75
Hydrothermal/solvothermal	High crystallinity, tunable morphology	Phase & shape control	Slow, autoclave-bound	21, 78 and 101
Sonochemical/microwave	Small, uniform nanocrystals	Rapid nucleation	Specialized equipment	76 and 87
Green/biosynthesis	Capped NPs, added functionality	Low toxicity & cost	Reproducibility, size control	44, 102 and 103
*In situ*/impregnation	Built-in heterointerfaces	Strong support integration	Interface control	85 and 91
Electrodeposition	Supported films	Binder-free, scalable	Under-explored for photocatalysis	104

Co-precipitation is the simplest and most scalable but often yields poorly crystalline, agglomerated particles requiring post-synthesis annealing.^71,75^ Hydrothermal and solvothermal methods, conducted in sealed autoclaves, afford superior control of phase purity, crystallinity, and morphology nanorods, nanoflowers, and microspheres and dominate the recent literature.^21,78,101^ Sonochemical and microwave-assisted routes accelerate nucleation, giving small, uniform nanocrystals in short reaction times.^76,87^ Green and biosynthetic approaches, using plant extracts or microorganisms as reducing and capping agents, offer low-toxicity, low-cost synthesis and frequently confer additional antibacterial functionality, though batch-to-batch reproducibility and precise size control remain challenging.^44,102,103^*In situ* composite formation and wet-impregnation–calcination integrate selenides with carbon, polymer, or oxide supports, building the heterointerfaces central to charge separation.^85,91^ Electrodeposition, well established for selenide films in photovoltaics, remains under-explored for photocatalytic powders and represents an opportunity for scalable, binder-free, electrode-supported catalysts.

### 4.2 Physicochemical characterization

**Fig. 4:**
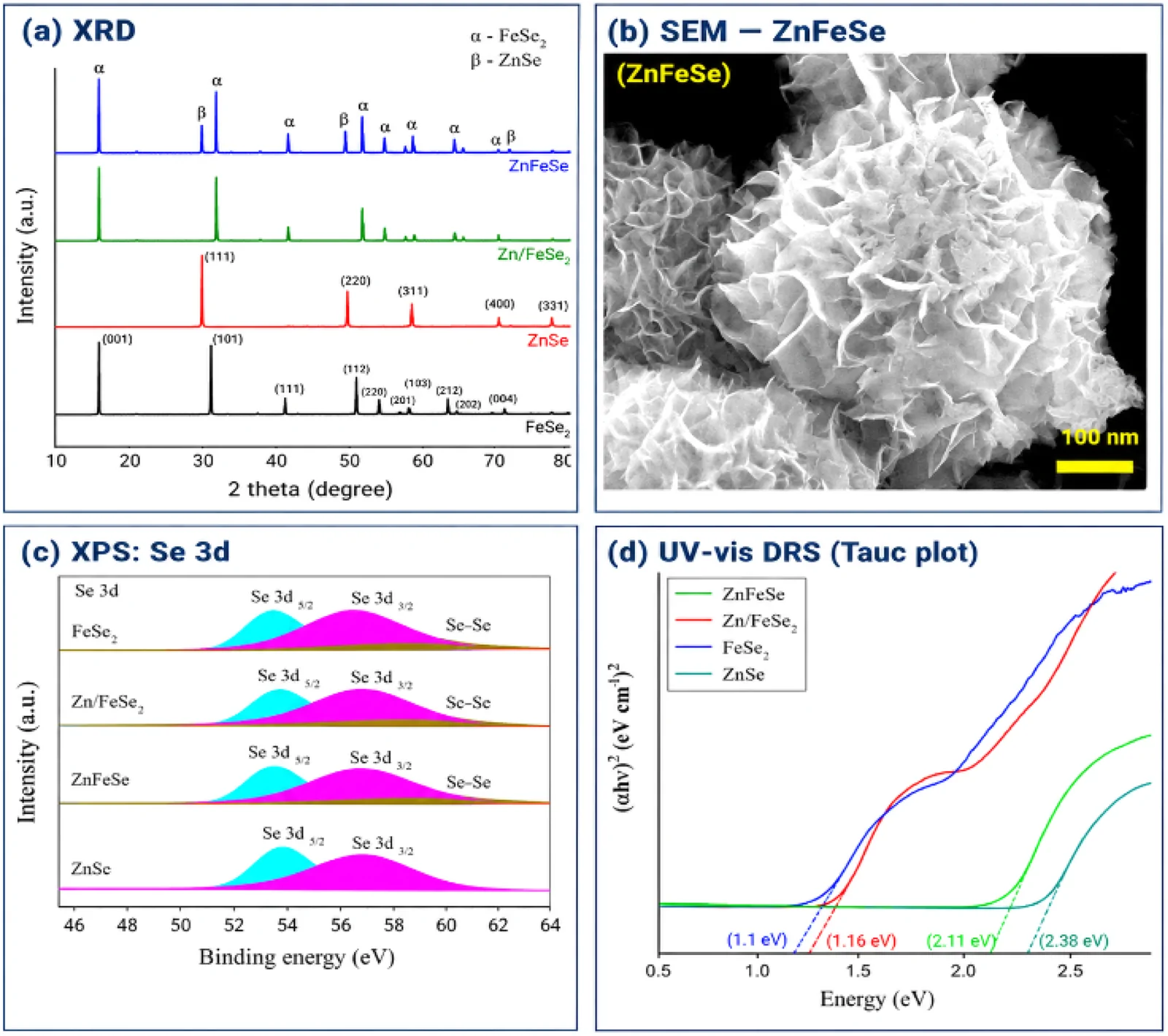
Representative characterization of metal selenide photocatalysts (FeSe_2_, ZnSe, Zn/FeSe_2_, ZnFeSe): (a) XRD, (b) SEM, (c) Se 3d XPS, (d) UV-vis DRS Tauc plots, with permissions.^105^

Reliable structure–property analysis rests on a standard characterization toolkit, with representative data illustrated in Fig. 4.

X-ray diffraction (XRD) establishes phase purity, crystallite size (Scherrer analysis), and lattice strain, and tracks the phase changes induced by annealing. Electron microscopy (SEM/TEM) resolves particle size, morphology, and interfacial contact, while HRTEM lattice fringes and selected-area diffraction confirm crystallinity and heterojunction quality. X-ray photoelectron spectroscopy (XPS) yields the oxidation states and surface composition of metal and selenium and is diagnostic of dopant incorporation, selenium vacancies, and metal–support charge transfer. UV-visible diffuse-reflectance spectroscopy (UV-vis DRS) with Tauc analysis quantifies the optical band gap underlying visible-light absorption, and photoluminescence probes charge-carrier recombination. Nitrogen adsorption–desorption (BET/BJH) determines surface area and porosity, and hence the density of accessible active sites. Together these techniques link synthesis to the properties governing photocatalytic performance.

### 4.3 Structure–property relationships

**Fig. 5:**
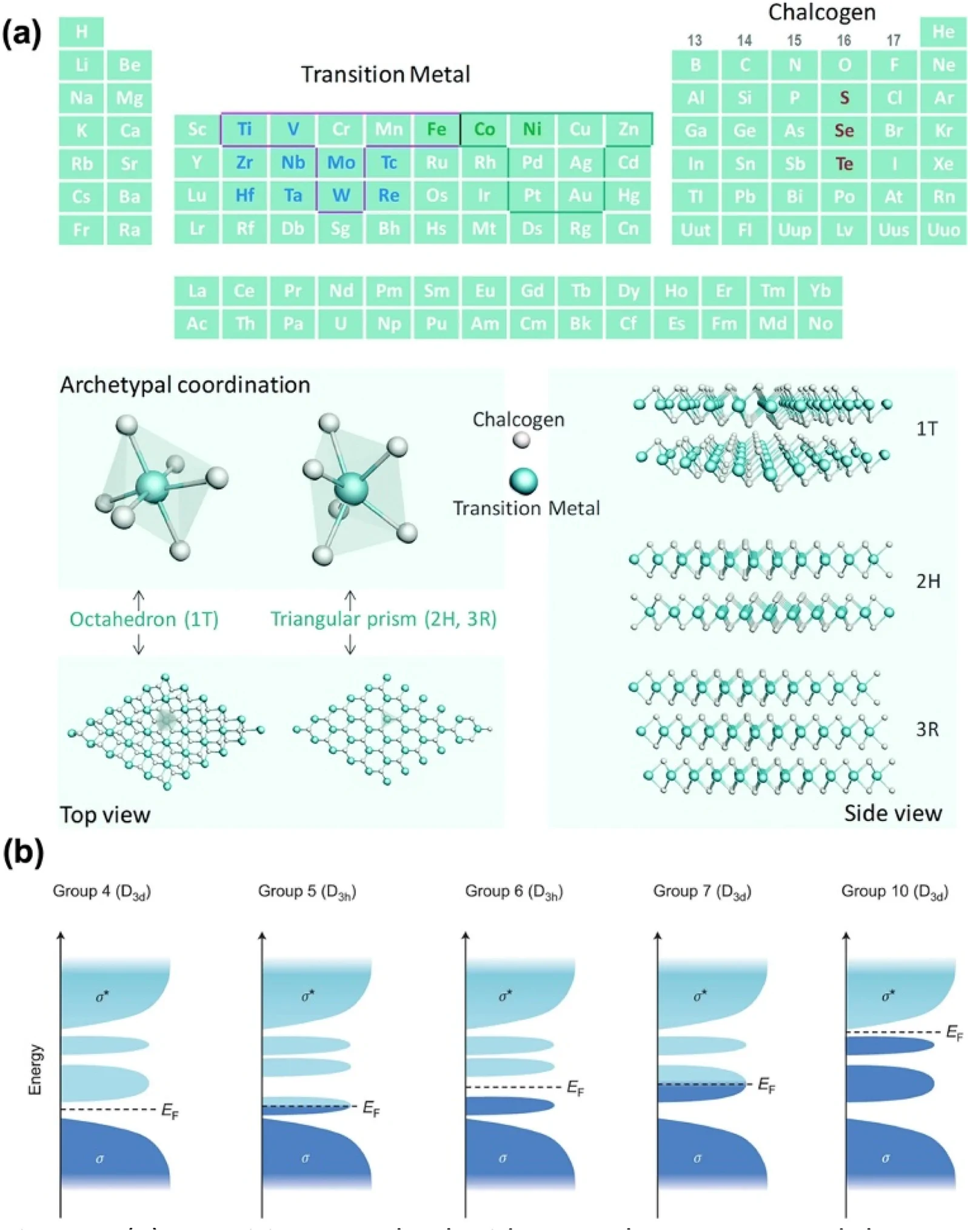
(a) Transition-metal-selenide crystal structures and the typical 1T, 2H, and 3R polytypes (top and side views); (b) d-orbital filling of various TMSes. Reproduced with permission from Dolla *et al.*, *Coordination Chemistry Reviews*, 2026, **559**, 217792 (DOI: https://www.doi.org/10.1016/j.ccr.2026.217792). Copyright 2026 Elsevier B.V.^107^

Photocatalytic performance is governed less by composition alone than by the microstructural features synthesis imparts as summarised in Fig. 5. Reducing particle size increases specific surface area and shortens the carrier diffusion path to the surface, raising activity but at very small sizes quantum confinement widens the band gap and can blue-shift absorption out of the visible, so an optimum size exists. Morphology dictates the exposed facets and accessible surface: high-surface-area nanorods, nanoflowers, and porous microspheres consistently outperform bulk or agglomerated particles. Crystallinity is a recurring determinant the improvement of ZnSe activity on annealing from 200 to 800 °C, and the bandgap narrowing that accompanies impurity removal in annealed copper selenides, both show how higher crystallinity reduces bulk recombination centres.^75,106^ Controlled defects, particularly selenium vacancies, act as shallow traps and adsorption sites that enhance charge separation and O_2_ activation, though excessive defect density becomes recombination–promoting a balance, not a monotonic benefit. Dopant concentration behaves similarly: moderate doping narrows the gap and traps carriers beneficially, whereas excess dopant introduces recombination centres, as seen where increasing CoSe content in ZnSe/CoSe ultimately reduced activity.^94^ Finally, heterointerface quality intimate, defect-free contact between phases determines how effectively the charge-transfer mechanisms of Section 7 operate; poor contact negates an otherwise favourable band alignment. These relationships convert the empirical performance trends of later sections into transferable design rules.

## 5. Organic dyes as pollutants

**Fig. 6:**
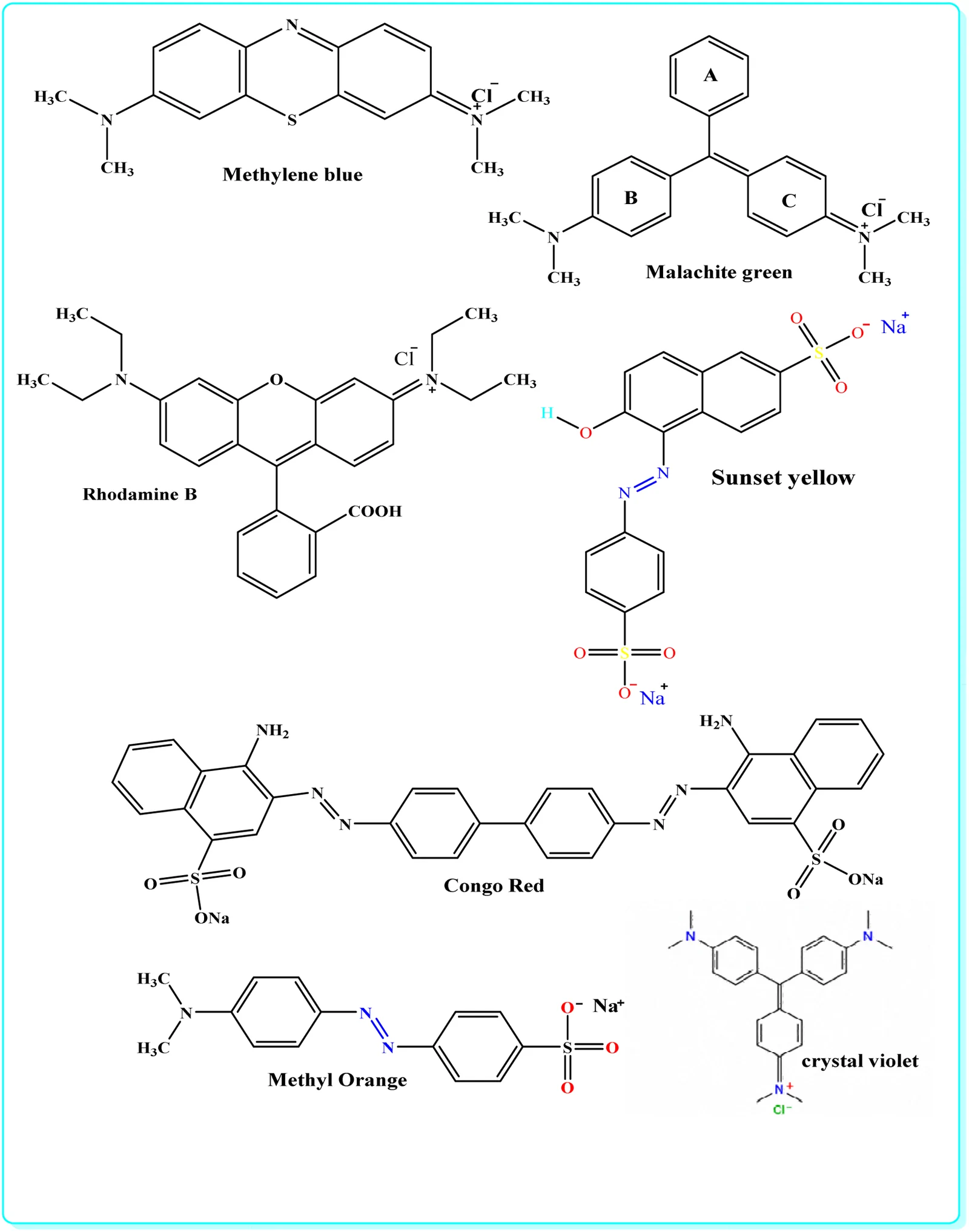
Chemical structures of representative organic dyes degraded by metal selenide photocatalysts: methylene blue, malachite green, rhodamine B, sunset yellow, Congo red, methyl orange, and crystal violet.

Dyes are complex organic compounds Fig. 6 that are often toxic, carcinogenic, and resistant to biodegradation. They possess conjugated aromatic structures containing chromophoric and auxochromic groups, which are responsible for their intense coloration. These compounds are primarily used to impart color to fabrics.^108^ Over the past decades, water and soil pollution has become a major global environmental concern, largely due to the widespread use of hazardous dyes in industries including textiles, cosmetics, paper, food processing, and pharmaceuticals.^109^ The dyes present in wastewater adversely affect the aquatic ecosystem because the intense colour of the aqueous body imparted by dyes inhibits the access of sunlight to the interior of the aqueous body. Dyes have complex structures.^16^ The aromatic arrangements of dyes imparts resistance to photodegradation, thermal breakdown, biodegradation, and oxidative agents.^110^ The removal of dyes from wastewater is often prioritized over other organic contaminants, as even minute concentration of dyes (below 1 ppm) are visually detectable and can significantly impact aquatic environments.^111^ Dyes can be categorized as cationic, non-ionic and anionic.^112^

Direct comparison of the studies surveyed below is complicated because reported degradation efficiencies (% removal) depend strongly on the operating conditions catalyst dosage, initial dye concentration, light source and intensity, irradiation time, solution pH, reactor geometry, and the extent of dark adsorption. A catalyst reported at 99% removal is not necessarily superior to one at 85% if it used a higher dosage, a lower dye concentration, or a longer irradiation time. Wherever the primary studies permit, performance is therefore compared here on a kinetic basis: the apparent pseudo-first-order rate constant (*k*_app, min^−1^), which most dye-degradation systems obey, is a more transferable descriptor than percentage removal, particularly when normalized to catalyst mass or surface area (min^−1^ g^−1^ or min^−1^ m^−2^). Quantum efficiency and total-organic-carbon (TOC) removal, needed for fully rigorous benchmarking, are reported in only a minority of studies and remain a key gap in this field.

### 5.1 Cationic dyes

Basic cationic dyes dissociate in water to produce positively charged colored ions. Their molecular structures often feature functional groups such as azo, phthalocyanine, polycarboxylic, and solvent dye moieties. Despite their widespread industrial use, these dyes can be harmful to human health, potentially causing skin irritation, genetic alterations, and even cancer.^113^

#### 5.1.1 Methylene blue dye

Methylene blue (methylthioninium chloride) is a synthetic thiazine dye characterized by its high-water solubility and intense blue coloration. Widely used in textiles, paper, pharmaceuticals, and biomedical applications. It also serves as a pH/redox indicator and histological stain. Despite its versatility, methylene blue is environmentally hazardous, exhibiting carcinogenic, mutagenic, and neurotoxic effects, along with respiratory, ocular, and gastrointestinal toxicity upon excessive exposure.^52,53^ Methylene blue is an aromatic heterocyclic cationic thiazine dye (C_16_H_18_N_3_ClS, *M* = 319.85 g mol^−1^) with a characteristic maximum absorption at 663 nm. It is chemically identified as 3,7-bis(dimethylamino)phenothiazine chloride (CI 52015), and its molecular structure is shown in Fig. 6.^114^ A wide range of metal selenide-based photocatalysts has been explored for the efficient degradation of organic dyes, particularly methylene blue, under UV and visible-light irradiation. Sridevi *et al.*^71^ synthesized CdSe and ZnSe nanoparticles *via* co-precipitation and hydrothermal routes, employing mercaptoethanol and hydrazine hydrate as stabilizing agents. Both materials demonstrated effective photodegradation of methylene blue under UV light, following pseudo-first-order kinetics, with variations in performance attributed to differences in band gap energy and particle size. To further enhance photocatalytic efficiency under visible light, Mosavi *et al.*^115^ developed an Ag@CdSe/zeolite nanocomposite, achieving approximately 90% degradation of methylene blue along with excellent stability and recyclability. The incorporation of zeolite supports, combined with response surface methodology (RSM) optimization, proved highly effective for wastewater treatment applications. Similarly, Hsieh *et al.*^86^ reported ZnSe/graphene nanocomposites synthesized *via* a hydrothermal approach, where graphene oxide was integrated with ZnSe precursors. The resulting nanocomposite exhibited remarkable photocatalytic performance under visible light, achieving over 85% degradation within 1 h and up to 99.5% after 4 h. In another study, Patel *et al.*^75^ prepared Cu_1.8_Se nanoparticles through a co-precipitation method followed by annealing at 200 °C. The annealed samples exhibited a blue-shifted band gap (2.0–2.1 eV), attributed to impurity removal and improved crystallinity, which in turn enhanced photocatalytic degradation efficiency due to increased surface area and structural stability.

Optimization strategies have also been widely investigated. Gharbani *et al.*^116^ optimized CdSe nanoparticles for visible-light-driven degradation of methylene blue, achieving 92.8% predicted and 90.87% experimental efficiency under optimal conditions. However, reusability studies over ten cycles revealed a decline in performance from 89.87% to 58.76%, primarily due to catalyst loss and blockage of active sites by intermediate species, highlighting challenges associated with long-term stability. Likewise, Patel *et al.*^117^ synthesized cubic-phase Cu_2−*x*_Se nanoparticles with a direct band gap of ∼2.1 eV, demonstrating enhanced photocatalytic activity under solar irradiation. The degradation efficiency increased with catalyst loading, reaching 90.3% at optimal dosage, while maintaining reasonable stability over multiple cycles despite minor losses due to surface fouling and operational variations.

Beyond single-phase systems, compositional tuning has shown significant potential. Patel *et al.*^76^ synthesized Cu_*x*_Sn_1−*x*_Se nanocrystals *via* an ultrasound-assisted method, achieving efficient degradation of methylene blue and crystal violet under UV irradiation. Notably, SnSe exhibited superior performance, achieving 98% and 88.68% degradation, respectively, within 6 h, and retained high stability over six cycles. Similarly, Ashiq *et al.*^72^ reported SnSe nanostructures with tuneable Sn : Se ratios, demonstrating rapid and efficient visible-light-driven degradation, with efficiencies reaching up to 88.9% within 10 min, thereby highlighting their potential as cost-effective photocatalysts.

Green synthesis approaches have also gained attention. Velayutham *et al.*^44^ synthesized NiSe nanoparticles *via* a hydrothermal method using *Hibiscus rosa-sinensis* extract, achieving 92% degradation of methylene blue under simulated solar light. The enhanced performance was attributed to improved light absorption, suppressed electron–hole recombination, and efficient generation of reactive oxygen species (ROS). Additionally, the material exhibited notable antibacterial activity against *E. coli* and *S. aureus*, indicating its dual functionality in wastewater treatment and microbial control.

In parallel, hybrid and flexible systems have been developed to further enhance photocatalytic performance. Hameed *et al.*^91^ fabricated CNF/Ag_2_Se nanocomposite films *via in situ* synthesis, resulting in significant band gap narrowing (from 4.61 to 2.71 eV) and achieving 99% degradation under visible light due to improved charge separation and increased active sites. Similarly, Yin *et al.*^94^ reported ZnSe/CoSe nanocomposites with 99.4% degradation efficiency within 120 min, significantly outperforming pure ZnSe. This enhancement was attributed to synergistic band alignment, increased surface area, and efficient charge carrier separation, although excessive CoSe content led to reduced activity, emphasizing the importance of optimized composition.

Advanced heterostructures and carbon-based hybrids have also demonstrated superior performance. Oh *et al.*^87^ synthesized graphene–PtSe_2_ nanocomposites *via* a microwave-assisted method, exhibiting efficient degradation of methylene blue and rhodamine B under visible light, facilitated by π–π interactions, enhanced charge transfer, and radical formation. Similarly, Cheng *et al.*^95^ developed CuSe/MoSe_2_ 2D/2D heterojunctions, achieving 4.5-fold higher degradation efficiency and significantly improved photocurrent compared to pristine MoSe_2_, due to enhanced interfacial contact and charge transfer dynamics.

The role of graphene-based materials as electron mediators has also been extensively explored. Patidar *et al.*^98^ synthesized CdSe/rGO nanohybrids, where the incorporation of reduced graphene oxide significantly improved photocatalytic activity, achieving 70% degradation due to enhanced light absorption and suppressed electron–hole recombination. Likewise, Tian *et al.*^96^ reported MoSe_2_/ZnSe heterostructures with full-spectrum UV-visible absorption, achieving 91.5% degradation efficiency, attributed to their band-matched 0D/2D architecture that facilitated efficient charge separation.

Finally, Nouri *et al.*^89^ synthesized copper selenide nanostructures and Cu_3_Se_2_/rGO nanocomposites *via* a green co-precipitation method. The incorporation of rGO significantly enhanced visible-light absorption and photocatalytic activity, with Cu_3_Se_2_/rGO (15%) achieving 92% degradation within 1 h. The nanocomposites also demonstrated good stability over multiple cycles with minimal structural changes, underscoring their potential for practical wastewater treatment applications and are summarised in Table S1. A comparison of representative metal selenide photocatalysts for methylene blue degradation is summarized in Table 3, with the full dataset operating conditions, surface area, dominant ROS, and reusability provided in Table S5 (SI).

**Table 3:** Overview of methylene blue degradation by metal selenide-based photocatalysts (condensed). The full comparison, including operating conditions, surface area, dominant ROS, and reusability, is given in Table S9. References are given in SI

Photocatalyst	Synthesis route	*E*_g (eV)	Light source	% Degradation	*k*_app (min^−1^) [ref.]
CdSe & ZnSe NPs	Co-precipitation/hydrothermal	CdSe ∼2.1; ZnSe ∼2.6	UV	78	NR^71^
Ag@CdSe/zeolite	Facile synthesis	NR	Vis	89.98	NR^115^
ZnSe/graphene	Hydrothermal	NR	Vis	99.6	0.212 (ref. 86)
Cu_1_._8_Se NPs	Co-precipitation	NR	Incandescent	Max.	NR^75^
CdSe	Thermal treatment	2.55	Vis	90.87	0.038 (ref. 116)
Cu_2−*x*_Se NPs	Co-precipitation	2.1	Solar	90.30	NR^117^
Cu_*x*_Sn_1−*x*_Se	Sonochemical exfoliation	1.75–1.80	UV	88.68	NR^76^
SnSe	Chemical	NR	Vis	92.91	NR^72^
NiSe NPs	Hydrothermal	1.74	Vis	92	0.0189 (ref. 44)
CNF/Ag_2_Se film	*In situ* synthesis	2.71	Vis	99	0.076 (ref. 91)
ZnSe/CoSe	Hydrothermal	2.63	Vis	99.4	NR^94^
PtSe_2_/graphene	Microwave-assisted	2.0	Vis	Max.	0.022 (ref. 87)
CuSe/MoSe_2_	Hydrothermal	1.71	Hg/Xe lamp	98	NR^95^
MoSe_2_/ZnSe	Hydrothermal	NR	Hg lamp	91.5	0.447 (ref. 96)
Cu_3_Se_2_/rGO	Co-precipitation	1.9	Solar	97	NR^89^

#### 5.1.2 Rhodamine B

Rhodamine B (RhB), a xanthene-based dye, is highly water-soluble and exhibits a characteristic absorption peak at 553 nm shown in Fig. 6. Despite its widespread use in industrial dyeing and water-tracing applications, RhB poses significant environmental and health concerns due to its pronounced carcinogenicity, neurotoxicity, and potential to cause mucocutaneous irritation.^118,119^ Consequently, the development of efficient photocatalytic systems for its degradation has attracted considerable research attention.

In this context, Ge *et al.*^74^ synthesized TiSe_2_ nanoplates *via* a facile solution-based approach, achieving approximately 96% degradation of RhB under natural sunlight within 30 min. The enhanced performance, compared to commercial TiO_2_ (P25), was attributed to superior visible-light absorption and efficient charge separation, highlighting TiSe_2_ as a promising solar-driven photocatalyst.

Transition metal selenides have also shown promising activity under UV irradiation. Firdaus *et al.*^73^ synthesized CoSe nanoparticles *via* a precipitation method, achieving efficient RhB degradation through reactive oxygen species (ROS) generation and effective charge separation, following pseudo-first-order kinetics. However, a decline in efficiency at higher dye concentrations indicated the influence of mass transfer and active site availability. In another study, Ullah *et al.*^121^ prepared PtSe_2_–graphene/TiO_2_ nanocomposites (PGT3) using an ultrasonic-assisted method, achieving 70% RhB degradation under combined UV-visible irradiation within 180 min. The enhanced performance was attributed to the synergistic interaction between PtSe_2_ and graphene, where the 2D graphene framework facilitated π–π interactions and significant dye adsorption (∼40%) prior to irradiation, along with improved charge transport properties. Advanced heterostructured systems have further demonstrated improved photocatalytic efficiency. Xu *et al.*^97^ developed a 3D Fe_3_O_4_/ZnO/ZnSe heterostructure integrated into a magnetically fixed-bed reactor for continuous solar-driven photocatalysis. The sea urchin-like morphology promoted efficient ZnSe–ZnO charge separation, enabling 96% RhB degradation within 12 h, along with good stability and applicability for other pollutants, thereby indicating strong potential for scalable wastewater treatment. Furthermore, Ramezani *et al.*^77^ synthesized Bi_2_Se_3_ nanoparticles *via* a sonochemical method, demonstrating photocatalytic activity for RhB and methylene blue degradation under UV light. Additionally, CdS-coated Bi_2_Se_3_ thin films were employed in solar cell applications, highlighting the multifunctional nature of selenide-based materials. Summary of comparison of photocatalytic activity is summarized in Table 4 and comprehensive version in SI Table S7.

**Table 4:** Overview of rhodamine b degradation by metal selenide-based photocatalysts. The full comparison dye concentration, catalyst dosage, pH, surface area, dominant ROS, irradiation time, and reusability is provided in the corresponding table in the SI

Photocatalyst	Synthesis route	*E*_g (eV)	Light source	% Degradation	*k*_app (min^−1^)	Ref.
TiSe_2_	Chemical (TOP-free)	1.44	Xe lamp	96	NR	74
ZnSeO_3_	Green synthesis	2.40	Visible	99	NR	120
CoSe NPs	Precipitation	1.8	UV	Max.	0.0218	73
PtSe_2_–graphene/TiO_2_	Ultrasonic-assisted	2.20	UV/visible	70	3.7 × 10^−3^	121
Fe_3_O_4_/ZnO/ZnSe	Green synthesis	2.15	Solar	97.8	NR	97
Se nanorods	Green synthesis	3.55	UV	85	NR	122
Se nanorods	Green synthesis	4.30	UV	85	NR	123
Bi_2_Se_3_ NPs	Sonochemical	1.66	UV	93–98	NR	77
Se nanosheets	Biogenic synthesis	1.74	Visible	Complete	0.0048	124

#### 5.1.3 Crystal violet dye

Crystal violet (CV), a cationic triarylmethane dye (C_25_H_30_N_3_Cl; 407.98 g mol^−1^), exhibits a characteristic absorption maximum in the range of 589–594 nm. It is extensively utilized in Gram staining, as a pH indicator, and in textile dyeing and ink formulations. However, its widespread use is accompanied by significant toxicological concerns, including severe ocular irritation, potential irreversible corneal damage, respiratory distress, gastrointestinal complications, and progressive mucosal injury upon prolonged exposure.^125,126^ Moreover, as a prototypical cationic dye shown in Fig. 6, CV exhibits strong electrostatic interactions with negatively charged mammalian cell membranes, facilitating rapid cellular uptake and thereby amplifying its toxic effects.

In response to these environmental and health challenges, the development of efficient photocatalytic systems for CV degradation has gained increasing attention. Ahmad *et al.*^101^ synthesized iron–bismuth selenide–chitosan microspheres (BISe-CM) *via* a one-pot solvothermal method, demonstrating efficient degradation of crystal violet following first-order kinetics. Systematic optimization of key operational parameters including catalyst dosage, dye concentration, irradiation time, and pH along with recyclability assessments, confirmed the material's potential as a sustainable photocatalyst for water purification.

Similarly, Mahmood *et al.*^92^ developed copper selenide-polyaniline (CS-PANI) composites and evaluated their photocatalytic performance for crystal violet degradation. The enhanced activity was attributed to improved charge transfer characteristics and synergistic interactions within the composite structure. Furthermore, the study emphasized the broader applicability of CS-PANI in semiconducting devices and highlighted its potential for integration into advanced heterojunction systems, such as TiO_2_- or WO_3_-based composites, for improved solar-driven photocatalytic performance. Summary of the photocatalytic activities compared with various catalytic systems are summarized in Table 5 and the comprehensive version in Table S3.

**Table 5:** Overview of crystal violet degradation by metal selenide-based photocatalysts. The full comparison dye concentration, catalyst dosage, pH, surface area, dominant ROS, irradiation time, and reusability is provided in the corresponding table in the SI

Photocatalyst	Synthesis route	*E*_g (eV)	Light source	% Degradation	*k*_app (min^−1^)	Ref.
BISe-CM	Solvothermal	2.68	Solar	99.04	0.014	101
CS–PANI	Chemical precipitation	2.74	Visible	89.5	NR	92

#### 5.1.4 Malachite green

Malachite green (MG) Fig. 6, an *N*-methylated diaminotriphenylmethane dye, appears as green crystalline solids with metallic luster and is widely used in dye industries due to its low cost and high efficacy. However, MG is highly toxic and banned in many countries yet remains in use because of its accessibility. It induces severe damage to hepatic, renal, branchial, and intestinal tissues, causes mammalian cell toxicity and is associated with carcinogenic, dermatological, and respiratory risks. Moreover, uncontrolled discharge of MG into aquatic system disrupts ecological balance, while its presence in irrigation water degrades soil quality and reduces crop productivity.^127,128^

Given these environmental and health concerns, significant efforts have been directed toward the development of efficient photocatalytic systems for MG degradation. Kulsi *et al.*^83^ synthesized Bi_2_Se_3_ and Ni-doped Bi_2_Se_3_*via* a solvothermal method, where Ni incorporation significantly enhanced visible-light activity. The doped catalyst achieved complete (100%) degradation of MG within 5 min (*k* = 1.21 min^−1^) in the presence of H_2_O_2_, markedly outperforming pristine Bi_2_Se_3_. Similarly, Ahmed *et al.*^129^ developed Ag–CdSe/GO nanocomposites embedded within cellulose acetate (Ag–CdSe/GO@CA) for MG degradation. The enhanced photocatalytic performance was attributed to synergistic interactions between Ag and graphene oxide, which facilitated efficient charge separation, while the cellulose acetate nanofiber matrix improved adsorption capacity and charge carrier transport. The heterostructured system also demonstrated excellent stability and reusability over five cycles, highlighting its potential as an efficient hybrid photocatalyst. The malachite green degradation by metal selenide photocatalysts is summarised in Table 6.

**Table 6:** Overview of malachite green degradation by metal selenide-based photocatalysts. The full comparison dye concentration, catalyst dosage, pH, surface area, dominant ROS, irradiation time, and reusability is provided in the corresponding table in the SI

Photocatalyst	Synthesis route	*E*_g (eV)	Light source	% Degradation	*k*_app (min^−1^)	Ref.
Ni-doped Bi_2_Se_3_	Solvothermal	NR	Visible	100	NR	83
Ag–CdSe@CA	Hydrothermal	NR	Visible	92–97	NR	129
rGO/SnSe	Hydrothermal	NR	Visible	95.5	NR	90
Se NPs	Green (eco-friendly)	NR	Sunlight	89	NR	102

To address the limitations of pristine selenides, Abdullah *et al.*^90^ synthesized reduced graphene oxide-supported SnSe (rGO/SnSe) nanocomposites *via* a hydrothermal approach. The composite exhibited significantly enhanced photocatalytic activity, achieving 95.5% MG degradation compared to 83.5% for pure SnSe. This improvement was attributed to enhanced charge transfer and suppression of electron–hole recombination facilitated by rGO. Additionally, the catalyst demonstrated good stability (86.5%), with photocatalysis primarily governed by efficient charge carrier dynamics. A comparison of the reported malachite green degradation studies is summarised in Table S4.

### 5.2 Anionic dyes

Anionic dyes are characterized by the presence of negatively charged functional groups typically sulfonate (–SO_3_^−^), carboxylate (–COO^−^), or phenolate (–O^−^) moieties covalently attached to their chromophoric backbone. These ionic substituents not only impart a net negative charge to the dye molecules in aqueous media but also significantly enhance their hydrophilicity, leading to high water solubility and mobility in wastewater streams.^130^ Structurally, anionic dyes encompass several major classes, including azo (–N=N–), anthraquinone (quinonoid), triphenylmethane, and nitro-based systems, each defined by distinct chromophoric groups responsible for their optical properties. Among these, azo dyes represent the largest and most widely utilized class, particularly in reactive dye formulations.^131,132^ Their prevalence arises from the synthetic versatility of the azo linkage, which allows extensive structural modification and the incorporation of multiple sulfonate groups, thereby improving solubility and fiber affinity. In textile applications, anionic azo dyes are often functionalized with reactive groups (*e.g.*, vinyl sulfone or chlorotriazine) that enable covalent bonding with fiber substrates, especially cellulose-based materials. However, their high solubility and chemical stability also render them persistent in aquatic environments, posing significant challenges for conventional wastewater treatment processes.^133^

#### 5.2.1 Methyl orange

Methyl orange MO, Fig. 6, an anionic sulfonated azo dye (C_14_H_14_N_3_NaO_3_S), exhibits high thermal and chemical stability (m.p. > 300 °C) and functions as a weak-acid pH indicator, displaying a distinct red-to-yellow transition within the pH range of 3.1–4.4. Its molecular structure consists of two benzene rings linked by an azo (–N=N–) bond, with one ring substituted by a dimethylamino group and the other by a sulfonic acid moiety Fig. 6.^134^ The presence of the azo linkage, sulfonate group, and extended π-conjugated system imparts intense coloration, significant toxicity, and resistance to biodegradation.^13^ Due to these properties, MO is extensively employed in textile, leather, and paper industries. However, its uncontrolled discharge into the environment poses serious ecological and health risks, including carcinogenic, mutagenic, and teratogenic effects. Furthermore, its persistence in irrigation systems contributes to soil degradation, reduced crop productivity, and overall loss of biodiversity, thereby necessitating efficient wastewater treatment prior to disposal.^135^

In this context, significant efforts have been directed toward developing advanced selenide-based photocatalysts for MO degradation. Chatterjee *et al.*^93^ synthesized polyaniline-Bi_2_Se_3_ (PANI–Bi_2_Se_3_) nanoflake composites *via* a combination of solvothermal growth and *in situ* polymerization. The optimized composite (PBS25) exhibited superior visible-light photocatalytic performance, attributed to enhanced charge separation and the generation of reactive oxygen species (˙OH and O_2_˙^−^ radicals), enabling rapid degradation of MO along with other dyes such as malachite green and rhodamine B. The material retained high stability, maintaining approximately 92–93% efficiency after four cycles. Similarly, Karuppasamy *et al.*^21^ developed ternary Zn_1−*x*_Ni_*x*_Se nanostructures *via* a one-pot hydrothermal method. Ni incorporation induced lattice distortion and bandgap narrowing, thereby enhancing visible-light absorption. The resulting porous nanostructures (70–90 nm) provided abundant active sites, while synergistic Zn–Ni interactions promoted efficient charge separation and suppressed electron–hole recombination. These features enabled superior degradation of methyl orange, Congo red, and chrome yellow, following pseudo-first-order kinetics, highlighting their scalability for wastewater remediation.

The influence of crystallinity and thermal treatment has also been investigated. Beena *et al.*^106^ synthesized ZnSe nanoparticles *via* co-precipitation followed by annealing at different temperatures (200–800 °C). The sample annealed at 800 °C exhibited enhanced photocatalytic activity (∼80% MO degradation) compared to the 200 °C sample (75%), primarily due to improved crystallinity, optimized band structure, and increased reactive oxygen species generation. Additionally, the nanoparticles demonstrated antibacterial activity against *E. coli* and *B. subtilis*, indicating their dual functionality. Further advancements have been achieved through heterostructure engineering. Yang *et al.*^88^ reported ZnO/ZnSe/MoSe_2_ ternary heterojunctions as efficient visible-light photocatalysts, achieving 91.5% MO degradation along with a 4.97 log reduction of *E. coli*. The enhanced performance was attributed to improved light absorption, efficient charge carrier separation, and the generation of reactive radicals (O_2_˙^−^ and ˙OH), demonstrating their multifunctional environmental remediation capability. The dye degradation statistics of MO are summarized in Table 7 and the comprehensive version is Table S6.

**Table 7:** Overview of methyl orange degradation by metal selenide-based photocatalysts. The full comparison dye concentration, catalyst dosage, pH, surface area, dominant ROS, irradiation time, and reusability is provided in the corresponding table in the SI

Photocatalyst	Synthesis route	*E*_g (eV)	Light source	% Degradation	*k*_app (min^−1^)	Ref.
PANI–Bi_2_Se_3_	Solvothermal/*in situ*	1.35	Visible	96.31	NR	93
Zn_1−*x*_Ni_*x*_Se	Hydrothermal	2.58	Visible	79.1	NR	21
ZnSe NPs	Co-precipitation	NR	Visible	80 (800 °C)	NR	106
ZnO/ZnSe/MoSe_2_	Hydrothermal	NR	Visible	91.5	NR	88
Se–ZnS	Wet impregnation	3.30	UV	95.5	8.4 × 10^−3^	85

#### 5.2.2 Congo red dye

Congo red (CR, Fig. 6), an anionic azo dye (C_32_H_22_N_6_Na_2_O_6_S_2_; *M* = 696.68 g mol^−1^), was first synthesized by Paul Böttiger in 1884 as an early direct dye. It exhibits a linear symmetrical structure, with two phenyl rings connected by a diazo linkage, each bearing naphthalene units substituted with sulfonic and amino groups.^136^ Congo red (1-naphthalenesulfonic acid, 3,3′-(4,4′-biphenylenebis(azo))bis(4-amino-), disodium salt) is a synthetic anionic azo dye commonly applied in rubber, plastics, textile, paper, and dyeing industries.^137^

Congo red is predominantly released into the environment from textile, printing, leather, cosmetic industries, and biomedical laboratories. Exposure to CR poses significant health risks, including respiratory distress, nausea, vomiting, and diarrhoea in both humans and animals.^138^ Owing to its toxicity and persistence, strict environmental regulations mandate the removal or degradation of such hazardous dyes from industrial effluents prior to their discharge into natural ecosystems.^139^

In response to these concerns, a variety of metal selenide-based photocatalysts have been developed for the efficient degradation of Congo red under solar and visible-light irradiation. Khan *et al.*^5^ synthesized ternary metal selenide–chitosan microspheres (ZBiSe-CM), which exhibited outstanding photocatalytic performance, achieving 99% degradation of CR under sunlight while following first-order kinetics. The catalyst also demonstrated excellent reusability, maintaining high activity over five cycles, thereby highlighting its cost-effective and sustainable potential for wastewater treatment. Similarly, Yang *et al.*^78^ prepared FeNiSe nanoparticles supported on chitosan microspheres (FeNiSe-CHM) *via* a solvothermal approach. The composite achieved 99% degradation of CR within 100 min under sunlight at pH 6 (0.20 g catalyst, 60 ppm dye), following second-order kinetics, indicating its high efficiency in treating dye-contaminated wastewater.

In another study, Yasmin *et al.*^17^ synthesized AgInSbSe_3_ through a hydrothermal method, achieving 77.8% degradation of Congo red under sunlight while retaining stability over three cycles, demonstrating its effectiveness as a photocatalyst for organic pollutant removal. Complementarily, Ali *et al.*^80^ developed a bismuth–cobalt selenide tri-composite embedded within chitosan microspheres *via* solvothermal synthesis. The material achieved 85% degradation of CR under optimized conditions and maintained high activity over five reuse cycles, underscoring its applicability for industrial wastewater remediation. The role of compositional tuning and doping has also been explored to enhance photocatalytic efficiency. Kulsi *et al.*^82^ synthesized Bi_2_Se_3_ and 5 mol% Ni-doped Bi_2_Se_3_*via* a solvothermal route. Under visible-light irradiation in the presence of H_2_O_2_, the Ni-doped catalyst achieved 94% degradation of Congo red and 97% degradation of rhodamine B within 90 min, significantly outperforming pristine Bi_2_Se_3_ (35% CR and 90% RhB in 120 min). The catalyst also demonstrated excellent stability over multiple cycles. Furthermore, Yasmin *et al.*^81^ reported the synthesis of a quaternary metal selenide composite *via* a hydrothermal method, which achieved approximately 87% degradation of Congo red within 60 min under sunlight. The enhanced performance highlights the potential of multicomponent selenide systems for efficient visible-light-driven photocatalysis and wastewater treatment applications. Table 8 summarises the photocatalytic activity comparison of catalysts towards Congo red dye and the comprehensive version is included in Table S2.

**Table 8:** Overview of Congo red degradation by metal selenide-based photocatalysts. The full comparison dye concentration, catalyst dosage, pH, surface area, dominant ROS, irradiation time, and reusability is provided in the corresponding table in the SI

Photocatalyst	Synthesis route	*E*_g (eV)	Light source	% Degradation	*k*_app (min^−1^)	Ref.
ZBiSe-CM	Solvothermal	2.35	Solar	99.63	0.10	5
FeNiSe-CHM	Solvothermal	2.09	Sunlight	99	0.0426	78
AgInSbSe_3_	Hydrothermal	1.97	Sunlight	77.8	0.018	17
BCSN-CM	Solvothermal	2.48	Sunlight	85	0.015	80
Ni-doped Bi_2_Se_3_	Solvothermal	1.2	Visible	90	0.032	82
Quaternary selenide	Hydrothermal	2.01	Sunlight	86.9	NR	81

#### 5.2.3 Alizarin red dye

**Fig. 7:**
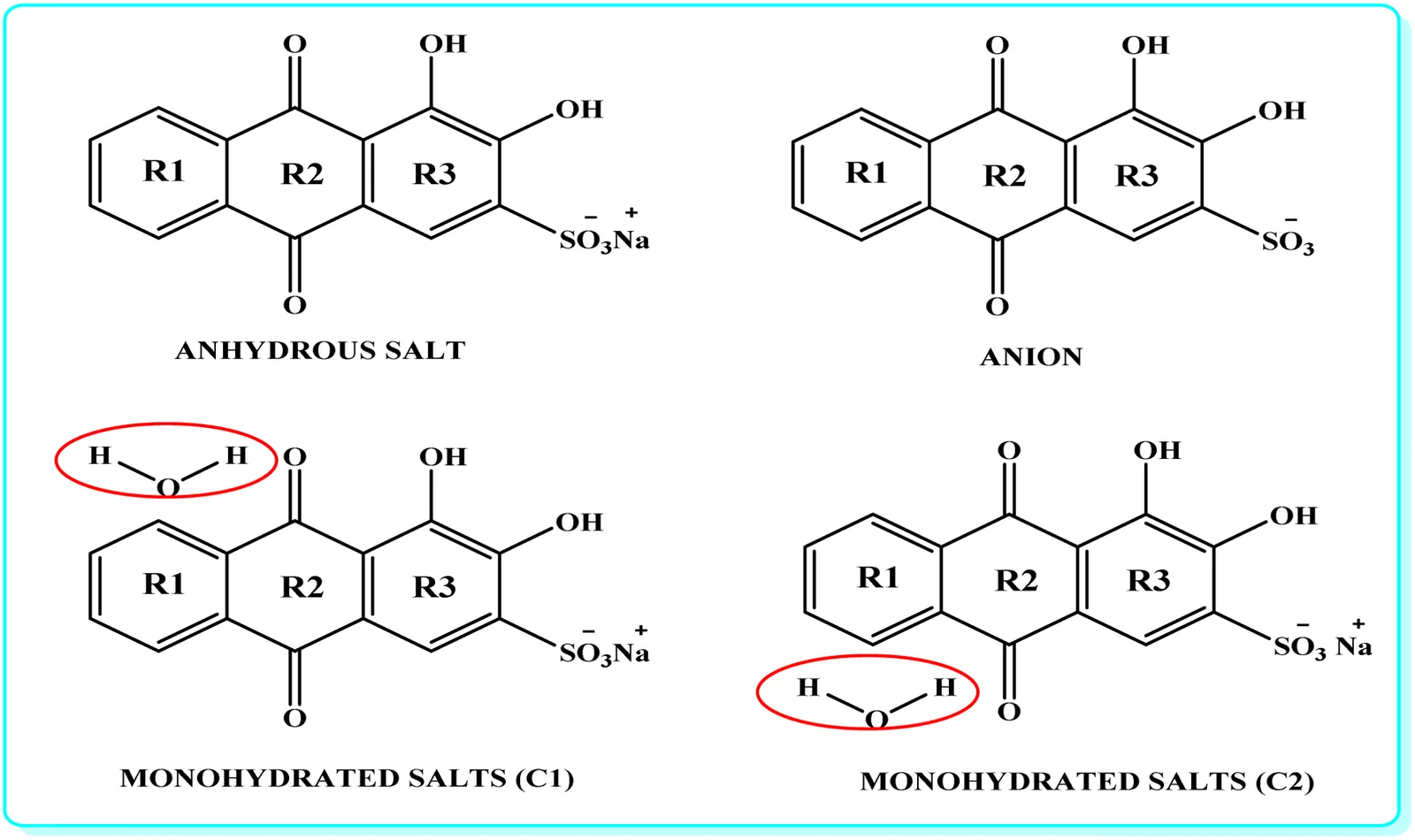
Structural representations of the anionic, anhydrous, and monohydrated sodium salts of alizarin red S, with ring designations and water molecules highlighted in red.^140^

Alizarin S (1,2-dihydroxyanthraquinone-3-sulfonate) Fig. 7 is a synthetic red anthraquinone dye, historically derived from madder roots and the first artificially synthesized dye as shown in Fig. 7. Widely used in textiles and biological staining, it exhibits carcinogenic and mutagenic effects, causing respiratory, ocular, and dermal toxicity, with a characteristic absorption maximum at 271 nm.^52^ In addition to conventional dye systems, metal selenide-based nanocomposites have also demonstrated significant potential for the degradation of other industrial dyes, such as Alizarin Red and Tartrazine. Azam *et al.*^45^ synthesized metal selenide–chitosan nanocomposites (MnNiSeC-NPs) *via* a co-precipitation route. The resulting nanocomposites exhibited efficient photocatalytic degradation of both dyes under sunlight, following pseudo-first-order kinetics. Systematic optimization of key operational parameters, including pH, catalyst dosage, dye concentration, and reaction time, confirmed their cost-effectiveness, reusability, and suitability for industrial wastewater treatment applications. Similarly, Ali *et al.*^1^ developed a ternary bismuth–copper selenide–chitosan microsphere nanocomposite (TMS-CM) with a well-defined spherical and porous morphology. The material achieved 86.7% degradation of Alizarin Red S over seven reuse cycles, following first-order kinetics. The enhanced performance, combined with excellent structural stability and recyclability, highlights its potential as a sustainable photocatalyst for dye-contaminated wastewater remediation as summarised in Table 9 and the comprehensive version is in Table S1.

**Table 9:** Overview of alizarin red s degradation by metal selenide-based photocatalysts. The full comparison dye concentration, catalyst dosage, pH, surface area, dominant ROS, irradiation time, and reusability is provided in the corresponding table in the SI

Photocatalyst	Synthesis route	*E*_g (eV)	Light source	% Degradation	*k*_app (min^−1^)	Ref.
MnNiSe–chitosan NPs	Co-precipitation	2.3	Sunlight	98	0.054	45
TMS–CM	Solvothermal	1.8	Sunlight	95.4	0.061	1

#### 5.2.4 Sunset yellow

Sunset Yellow (SY, Fig. 6)^143^ is an azo dye commonly used in foods, beverages, candies, desserts, and medicines, with a regulatory limit of 100–200 ppm.^141^ It improves visual appeal but excessive intake can cause hyperactivity, inattentiveness, and carcinogenic effects, highlighting the need for regulated use and effluent treatment.^103^ Metal selenide-based photocatalysts have also demonstrated significant potential for the degradation of food and industrial dyes such as tartrazine and sunset yellow. Zhang *et al.*^142^ synthesized chitosan–ZnSe (CS–ZnSe) nanoparticles *via* a chemical reduction method. Under optimized conditions, the nanocomposite achieved 98% and 97% degradation of tartrazine and sunset yellow, respectively, following first-order kinetics. The enhanced photocatalytic performance, coupled with excellent recyclability, underscores its potential for application in industrial wastewater treatment as summarised in Table 10 and the more comprehensive version is in Table S8.

**Table 10:** Overview of sunset yellow degradation by metal selenide-based photocatalysts. The full comparison dye concentration, catalyst dosage, pH, surface area, dominant ROS, irradiation time, and reusability is provided in the corresponding table in the SI

Photocatalyst	Synthesis route	*E*_g (eV)	Light source	% Degradation	*k*_app (min^−1^)	Ref.
CS–ZnSe NPs	Chemical reduction	NR	Sunlight	97	NR	142
Se-NPLs	Green synthesis	2.3	Solar/UV	83.8	0.173	103

## 6. Photocatalysis: fundamentals and thermodynamics

Advanced oxidation processes are widely employed for wastewater treatment, as strong oxidants efficiently degrade recalcitrant organic pollutants and remove select inorganic contaminants.^144,145^ Photocatalysis, as one of the AOPs,^41,146^ is a powerful technique that utilizes light-activated semiconductors to achieve rapid and complete degradation of organic contaminants under ambient conditions.^147^ Photocatalysis, a light-driven reaction in the presence of a semiconductor catalyst, has attracted attention due to its environmental compatibility, ability to fully mineralize pollutants, and no secondary pollution. The basic mechanism of photocatalysis is shown in Fig. 1.^70^ From a semiconductor photochemistry perspective, photocatalysis involves light-induced redox processes occurring within a semiconductor matrix. Semiconductors possess a characteristic electronic band structure comprising a lower-energy valence band (VB) and a higher-energy conduction band (CB), separated by an energy interval known as the band gap. Upon irradiation with photons of energy equal to or greater than the band gap, electrons are excited from the VB to the CB, generating electron–hole pairs. These charge carriers may subsequently migrate to the semiconductor surface under the influence of internal electric fields or concentration gradients. The photogenerated holes exhibit strong oxidative potential, enabling the oxidation of adsorbed species or dissolved substrates, while the excited electrons participate in complementary reduction reactions. Photocatalytic degradation is a light-driven process in which a semiconductor photocatalyst absorbs UV or visible light, generating electron–hole pairs. These charge carriers induce redox reactions that produce reactive oxygen species (ROS), including hydroxyl radicals (˙OH) and superoxide anions (O_2_˙^−^), which oxidize organic pollutants into less harmful products such as CO_2_, H_2_O, and inorganic ions. The process involves sequential steps of light absorption, charge carrier generation, ROS formation, and pollutant mineralization.^148–151^

**Fig. 8:**
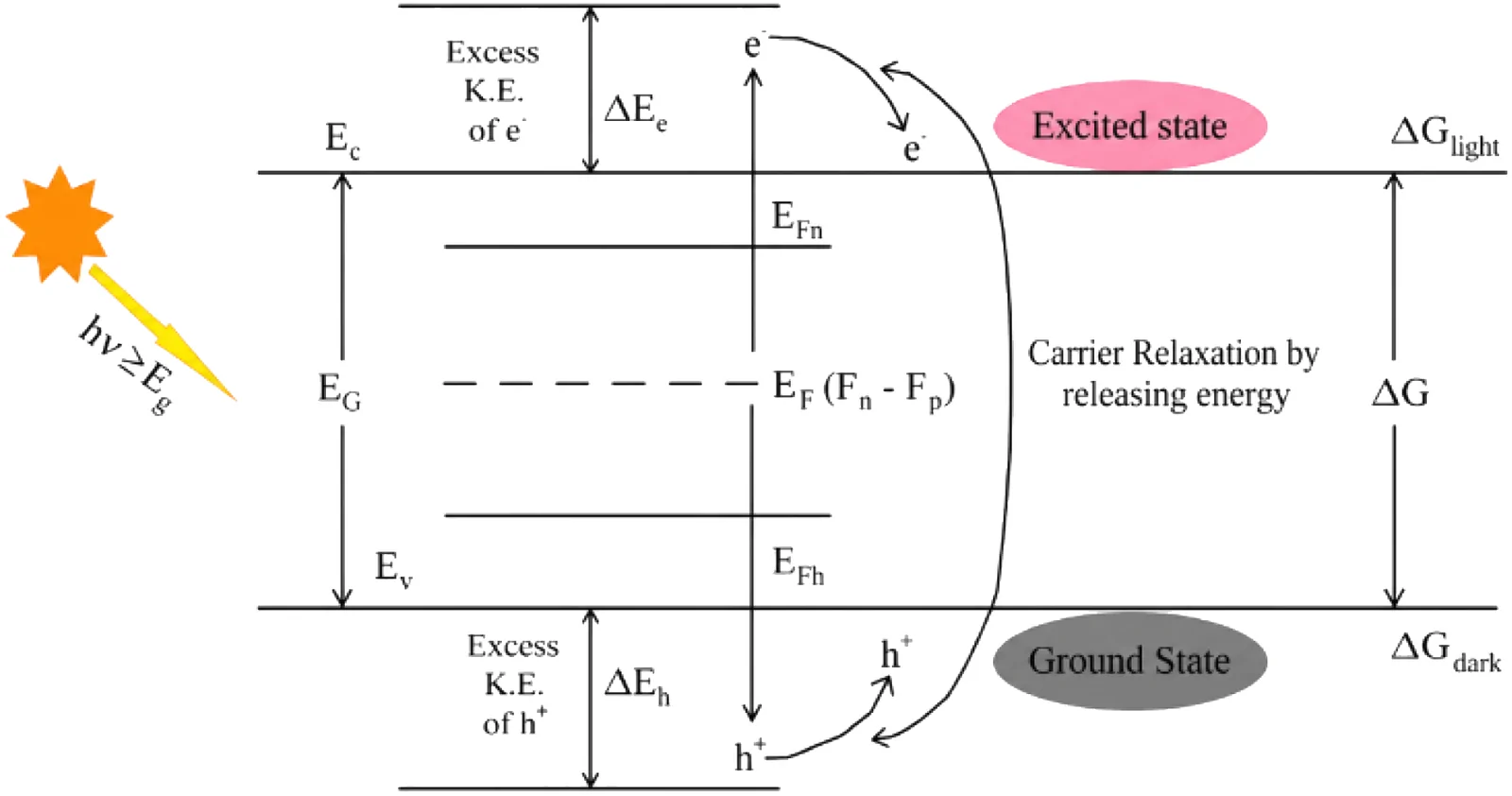
Thermodynamics along with Gibbs free energy (G) associated with a photocatalyst in the presence as well as absence of light^152^ Reproduced from Qanugo K., Bose D., Thakur K. K., *E3S Web of Conferences***309**, 01032 (2021), DOI: https://www.doi.org/10.1051/e3sconf/202130901032. Licensed under CC BY 4.0.

When a semiconductor absorbs light with energy greater than its band gap (E_9_), electrons are promoted from the valence band (VB) to the conduction band (CB), creating holes in the VB. These photogenerated charge carriers are mobile and can traverse the semiconductor lattice to participate in redox reactions. Due to rapid relaxation processes within the conduction band, electrons quickly attain an equilibrium energy state rather than recombining immediately across the band gap. Interactions with lattice vibrations facilitate the redistribution of electronic states, leading to a quasi-equilibrium condition. Under thermal equilibrium (Δ*H* = 0), the Gibbs free energy change (Δ*G*) becomes zero, resulting in no net driving force for a reaction. Therefore, in photocatalytic systems, the required Gibbs free energy is supplied by incident light, which drives the photochemical reactions as shown in Fig. 8.^152^

The degradation mechanism for different dyes as shown in Fig. 10–17. The aromatic structures are decomposed, ultimately converting the compounds into CO_2_ and H_2_O, thereby eliminating their colour and toxicity.

**Fig. 9:**
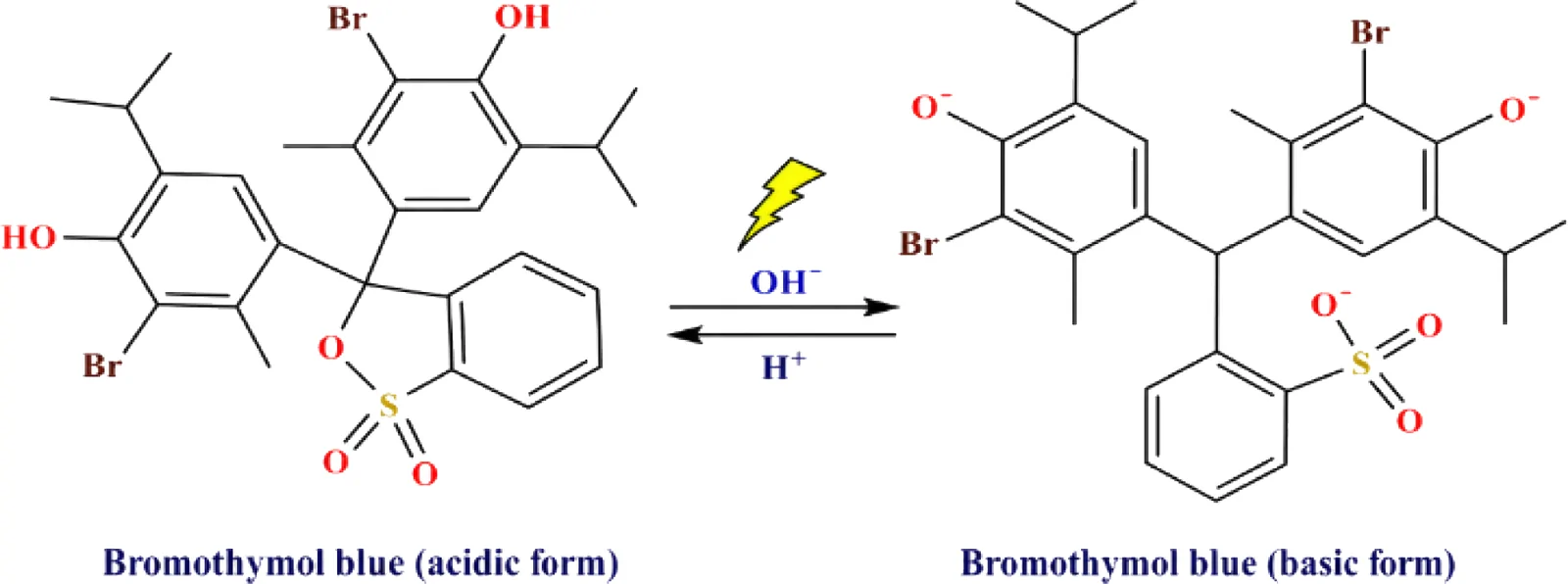
Chemical structures of the acidic (protonated) and basic (deprotonated) forms of bromothymol blue.

Bromothymol blue (BTB) is a sulfonphthalein dye widely employed as a pH indicator, exhibiting a distinct color transition from yellow (acidic form) to blue (basic form) within the pH range of approximately 6.0–7.6 as shown in Fig. 9.^22^

This color change arises from structural transformations between its protonated and deprotonated forms, involving changes in the conjugation and electron distribution across the molecule. In acidic conditions, the protonated form limits π-electron delocalization, whereas in alkaline media, deprotonation enhances conjugation, resulting in a bathochromic shift. The presence of electron-withdrawing groups such as bromine further stabilizes the molecular structure and influences its optical properties. Due to these characteristics, bromothymol blue serves as an important model compound for studying pH-responsive behavior and photocatalytic degradation mechanisms in aqueous systems.^153^

**Fig. 10:**
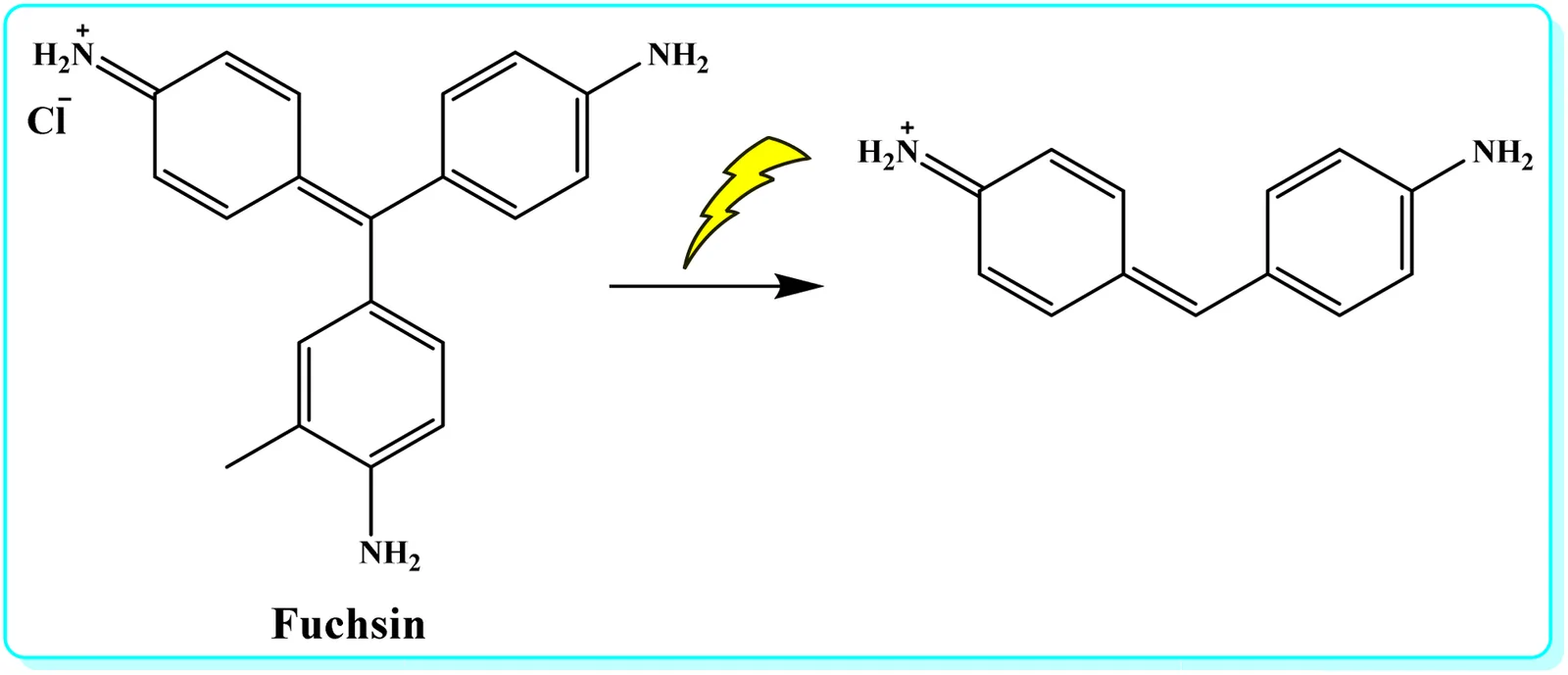
Schematic of the photocatalytic degradation of fuchsin and its primary breakdown product.^22^

Fuchsin is a triarylmethane dye widely used in biological staining, textile processing, and analytical applications due to its intense coloration and strong affinity for substrates as shown in Fig. 10.

**Fig. 11:**
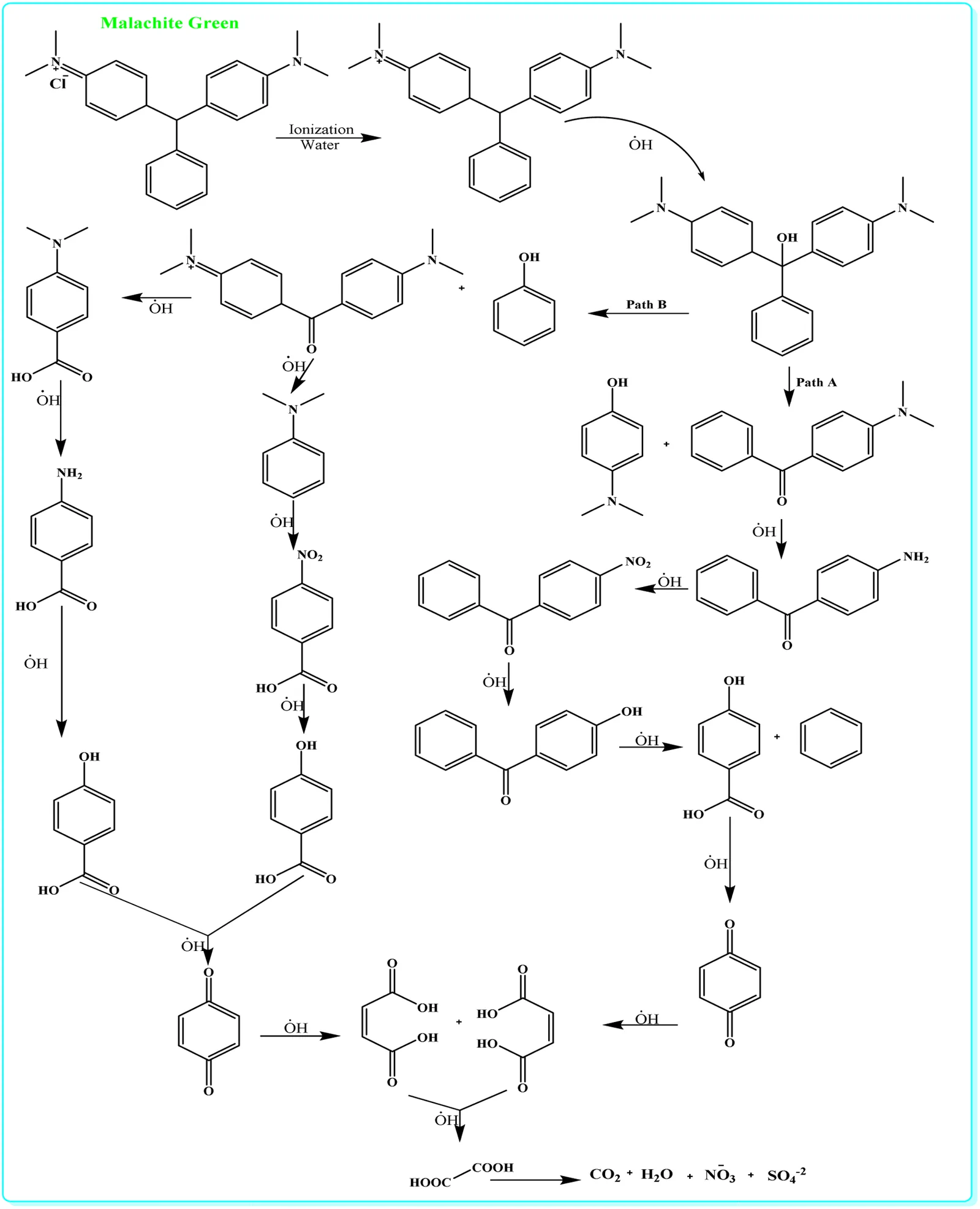
Schematic of photocatalytic degradation mechanism of malachite green.

Its chromophoric behaviour arises from an extended conjugated π-system, which facilitates visible-light absorption. Structurally, fuchsin exists in equilibrium with its reduced, colourless leuco form, where disruption of conjugation leads to loss of colour. This reversible transformation is highly sensitive to environmental conditions such as pH, light irradiation, and redox processes. In photocatalytic systems, degradation of fuchsin typically involves the breakdown of its conjugated framework through oxidative pathways, resulting in decolorization and eventual mineralization. Owing to its stability and toxicity, fuchsin is frequently employed as a model pollutant to evaluate the efficiency of photocatalysts in wastewater treatment.^154^ The compartmentalized schematic illustrates the stepwise photocatalytic degradation pathway of malachite green under solar irradiation in Fig. 11.^155^

Initially, the dye undergoes ionization and activation, followed by successive *N*-demethylation reactions induced by reactive oxygen species (˙OH and O_2_˙^−^), which progressively reduce the electron-donating character of the amine groups. Subsequent hydroxylation of the aromatic rings enhances molecular instability, facilitating cleavage of the central carbon framework and breakdown of the conjugated chromophore system.^156^ This leads to the formation of short-chain and open-chain intermediates, which are further oxidized into low-molecular-weight carboxylic acids. Ultimately, complete mineralization occurs, yielding environmentally benign end products such as CO_2_, H_2_O, NO_3_^−^, and SO_4_^2−^. The schematic highlights the sequential transformation from complex dye structure to simple inorganic species, emphasizing the efficiency of photocatalytic oxidation processes in wastewater remediation.^155,157^

**Fig. 12:**
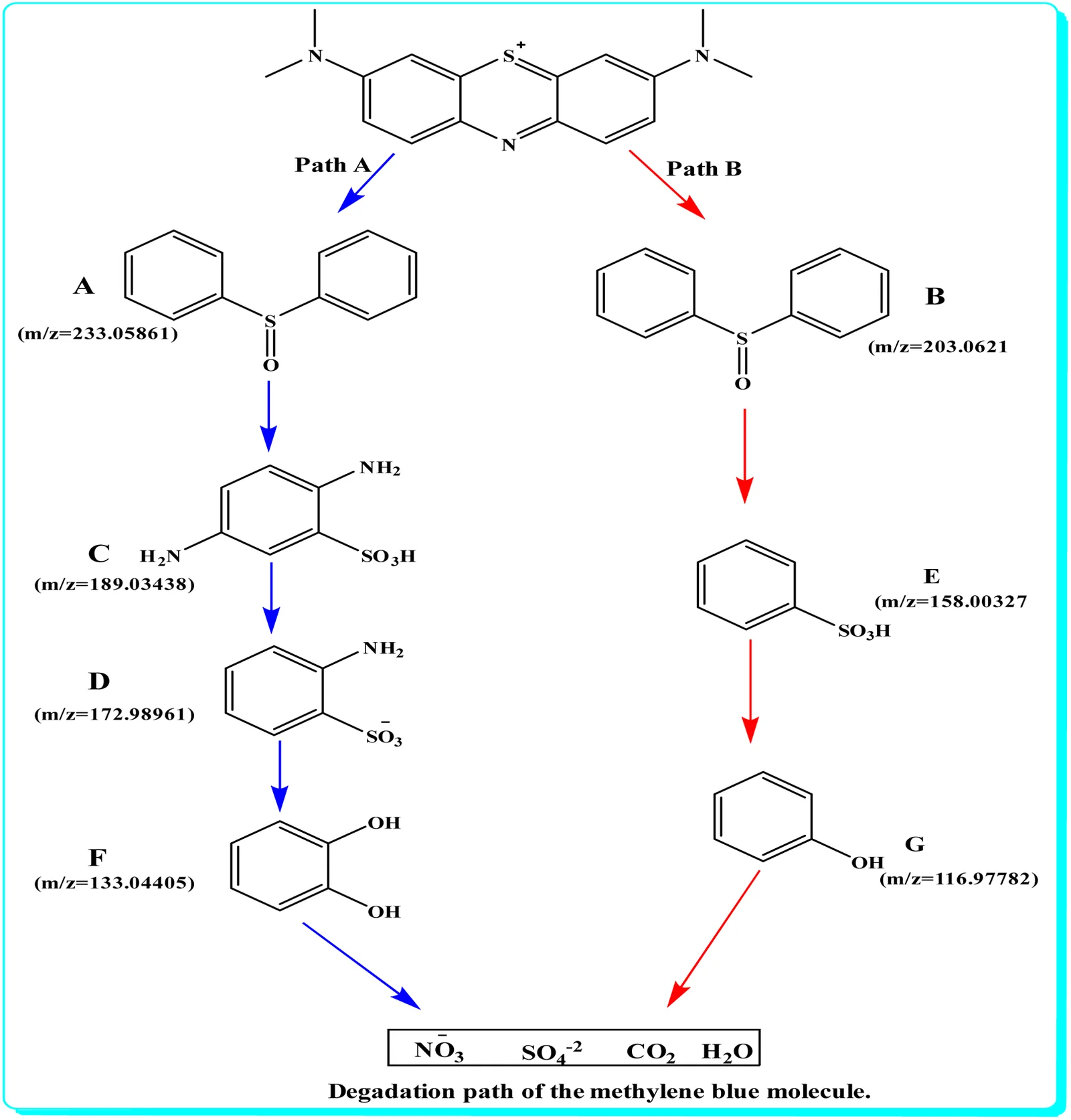
Proposed photocatalytic degradation pathway of methylene blue *via* two parallel routes (path A and path B), with intermediates identified by their *m*/*z* values.

The schematic presents the proposed photocatalytic degradation pathways of methylene blue through two parallel routes (path A and path B), as identified by intermediate species with corresponding *m*/*z* values shown in Fig. 12.^158^

Upon photoexcitation, the dye undergoes initial oxidative cleavage, leading to the formation of intermediate compounds *via* successive bond breaking and functional group transformations. In path A, stepwise oxidation and desulfurization processes generate aminated and sulfonated aromatic intermediates, which are further degraded into hydroxylated benzene derivatives. In path B, the degradation proceeds through sulfoxide formation and subsequent cleavage into simpler aromatic compounds such as benzenesulfonic acid derivatives, followed by further oxidation. Both pathways ultimately converge through progressive oxidation steps, resulting in ring-opening reactions and complete mineralization into inorganic end products, including NO_3_^−^, SO_4_^2−^, CO_2_, and H_2_O. This dual-path mechanism highlights the complexity and efficiency of photocatalytic processes in degrading methylene blue into environmentally benign species.^159,160^

**Fig. 13:**
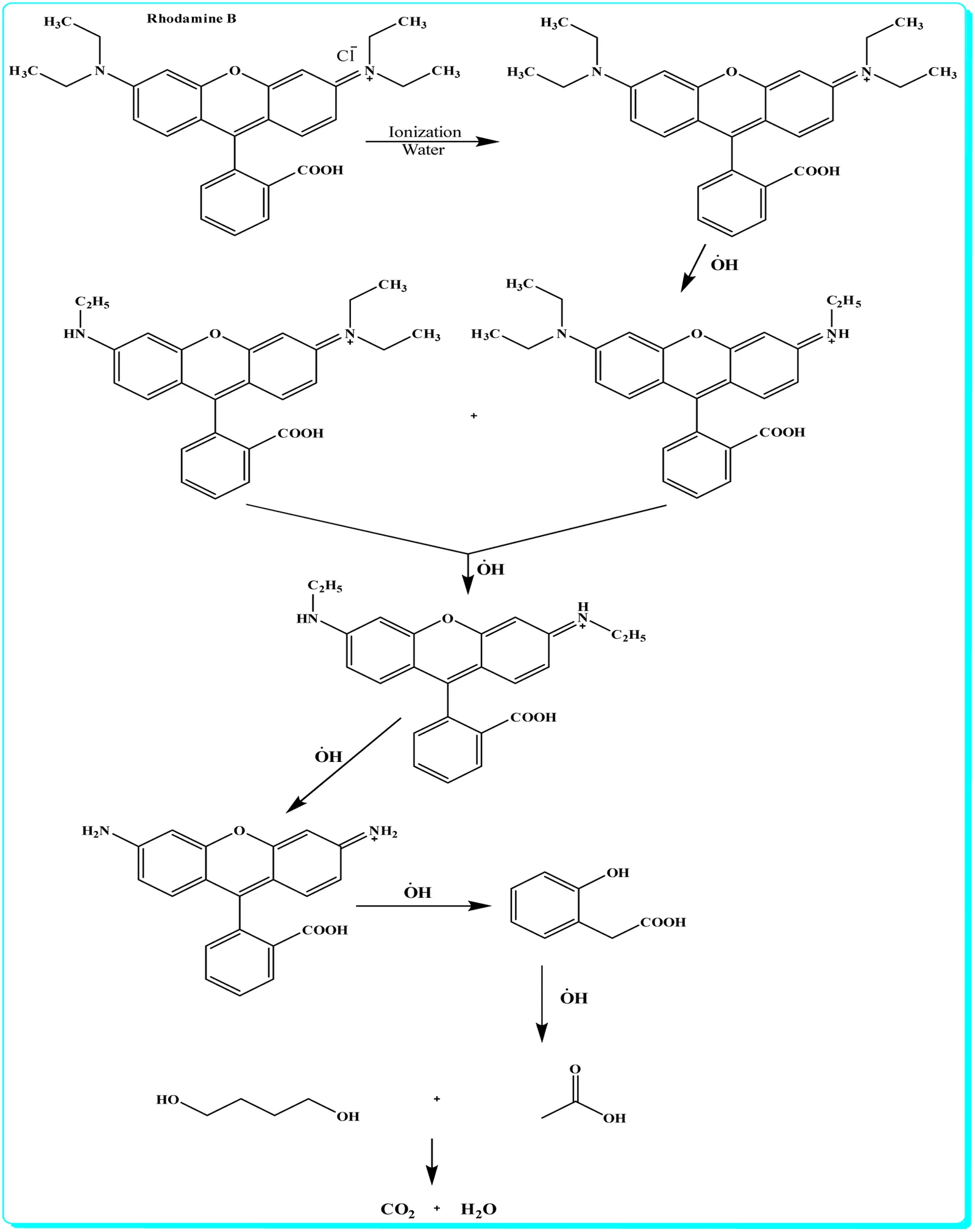
Schematic of photocatalytic degradation mechanism of rhodamine B.

The schematic in Fig. 13 illustrates the proposed photocatalytic degradation pathway of rhodamine B (RhB) under oxidative conditions. Initially, RhB undergoes ionization in aqueous media, followed by successive attacks by reactive oxygen species (˙OH), leading to stepwise *N*-deethylation of the ethyl groups attached to the amine functionalities.^155^ This process progressively reduces the electron-donating ability of the molecule and weakens its conjugated chromophore system. Subsequent hydroxylation reactions further destabilize the aromatic structure, facilitating cleavage of the xanthene ring and breakdown into smaller intermediate compounds, including carboxylic acid derivatives. Continued oxidation results in ring-opening reactions and formation of low-molecular-weight aliphatic species, which are ultimately mineralized into CO_2_ and H_2_O. This pathway highlights the sequential transformation of RhB from a complex, colored dye into simple, non-toxic end products through photocatalytic oxidation.^161^

**Fig. 14:**
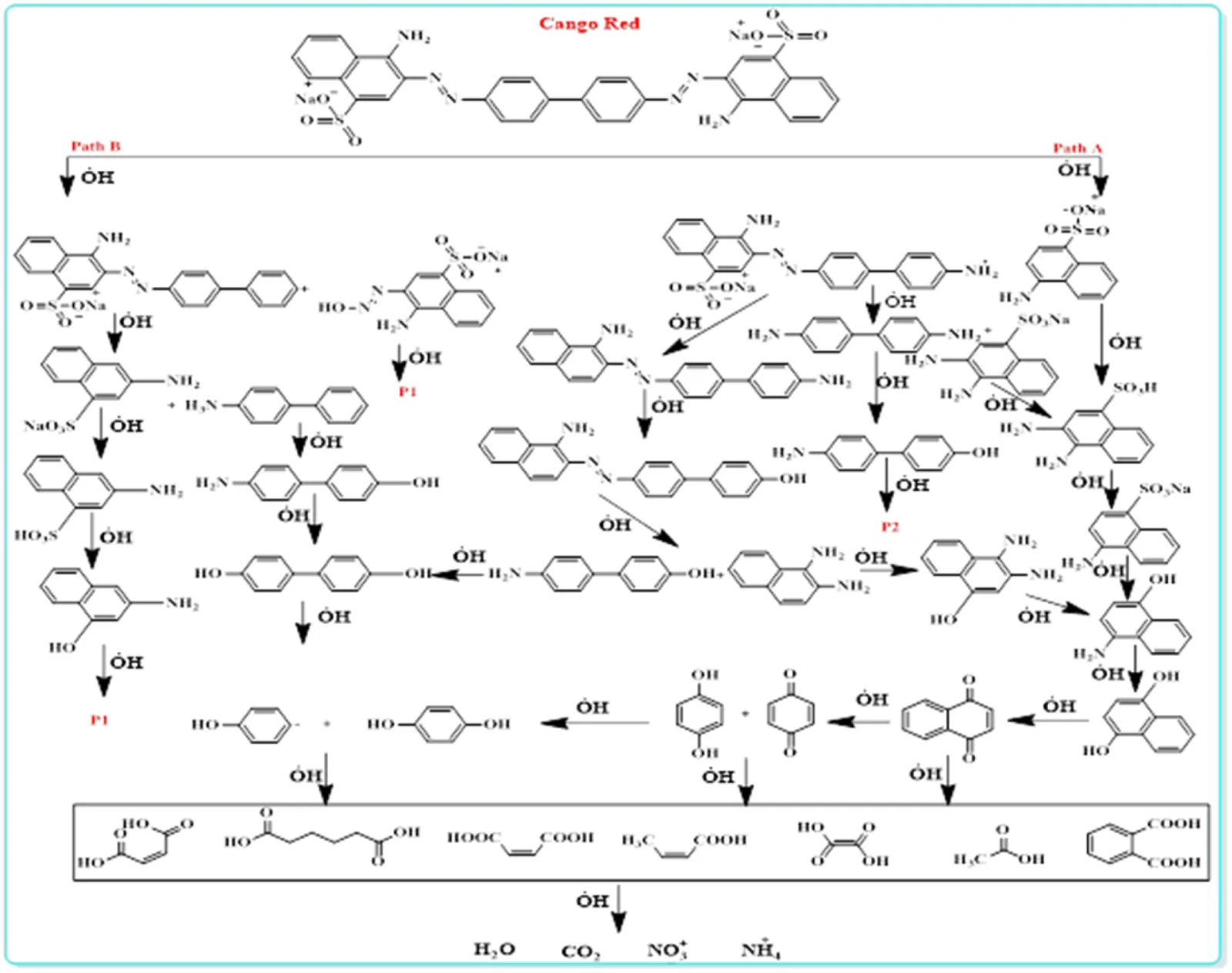
Schematic of photocatalytic degradation mechanism of Congo red.

The schematic diagram illustrates the proposed photocatalytic degradation pathways of Congo red under oxidative conditions, proceeding *via* two parallel routes: path A (˙OH attack on the azo bond) and path B (˙OH attack on the aromatic ring) as shown in Fig. 14.^155^

In path A, hydroxyl radicals initially cleave the azo (–N=N–) linkage, generating sulfonated aromatic intermediates and amine derivatives. These intermediates undergo further hydroxylation and oxidation, leading to the formation of smaller aromatic compounds such as phenols and carboxylic derivatives, followed by progressive ring cleavage. In path B, ˙OH radicals attack the naphthalene and benzene rings, resulting in direct breakdown of the aromatic structure into amine-containing intermediates, which are subsequently hydroxylated and degraded. Both pathways converge through successive oxidation steps, producing low-molecular-weight organic acids and ultimately achieving complete mineralization into inorganic end products such as CO_2_, H_2_O, NO_3_^−^, NH_4_^+^, and H_3_O^+^. This dual-path mechanism highlights the efficiency of photocatalytic systems in transforming complex azo dyes into environmentally benign species.^155,162,163^

**Fig. 15:**
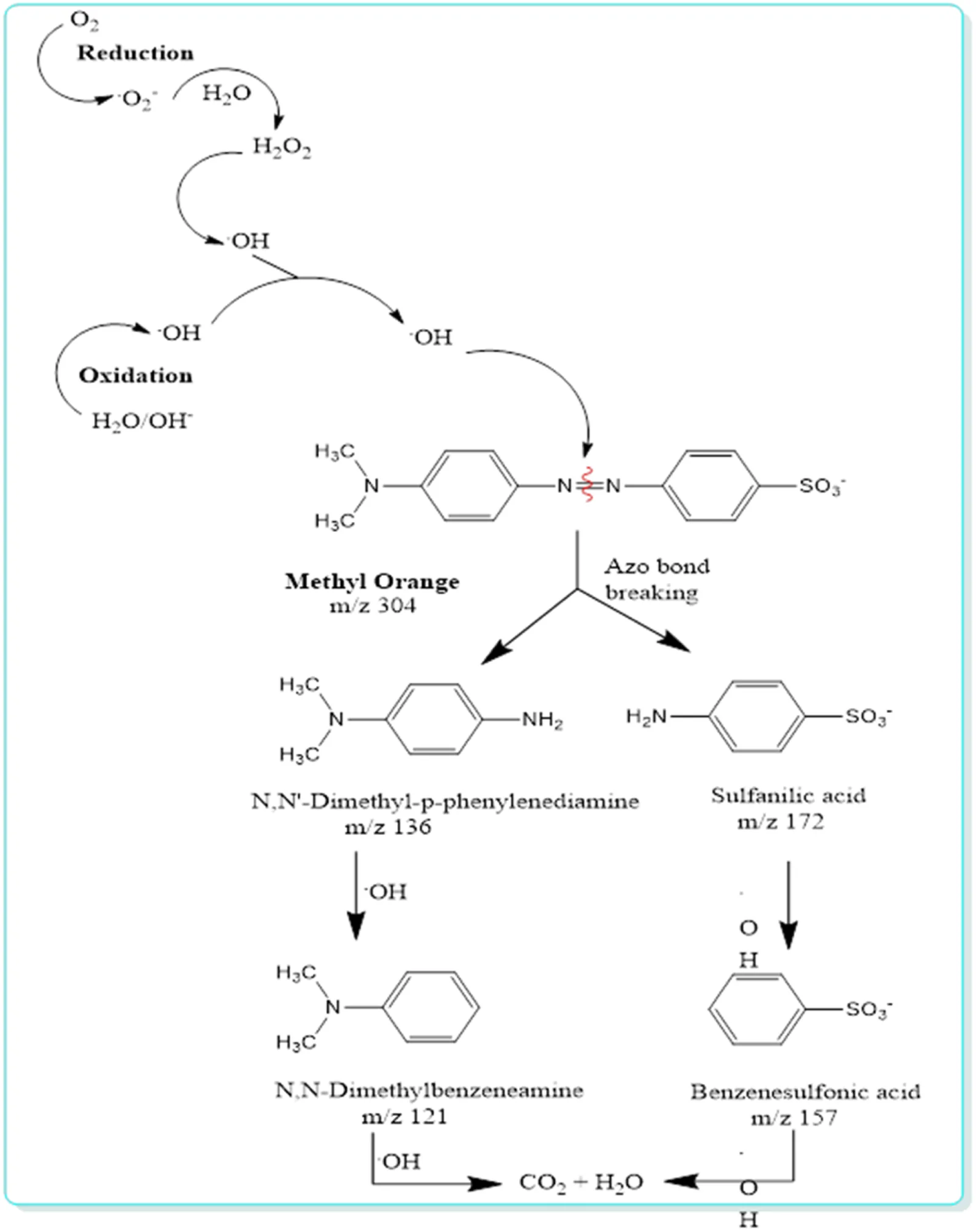
Schematic of the photocatalytic breakdown mechanism of methyl orange (MO) dye.

The schematic illustrates the photocatalytic degradation pathway of methyl orange (MO) initiated by the generation of reactive oxygen species (ROS), including superoxide (O_2_˙^−^), hydrogen peroxide (H_2_O_2_), and hydroxyl radicals (˙OH) as illustrate in Fig. 15.^134^

Upon irradiation, ˙OH radicals act as the primary oxidizing species, attacking the azo (–N=N–) bond of methyl orange (*m*/*z* 304), leading to its cleavage into key intermediates such as *N*,*N*-dimethyl-*p*-phenylenediamine (*m*/*z* 136) and sulfanilic acid (*m*/*z* 172). These intermediates undergo further oxidative transformations, including hydroxylation and deamination, producing simpler aromatic compounds such as *N*,*N*-dimethylbenzenamine (*m*/*z* 121) and benzenesulfonic acid (*m*/*z* 157). Continued oxidation results in the breakdown of aromatic structures and eventual formation of low-molecular-weight species, which are ultimately mineralized into CO_2_ and H_2_O. This pathway highlights the crucial role of hydroxyl radicals in initiating azo bond cleavage and driving complete degradation of methyl orange.^134,164,165^

**Fig. 16:**
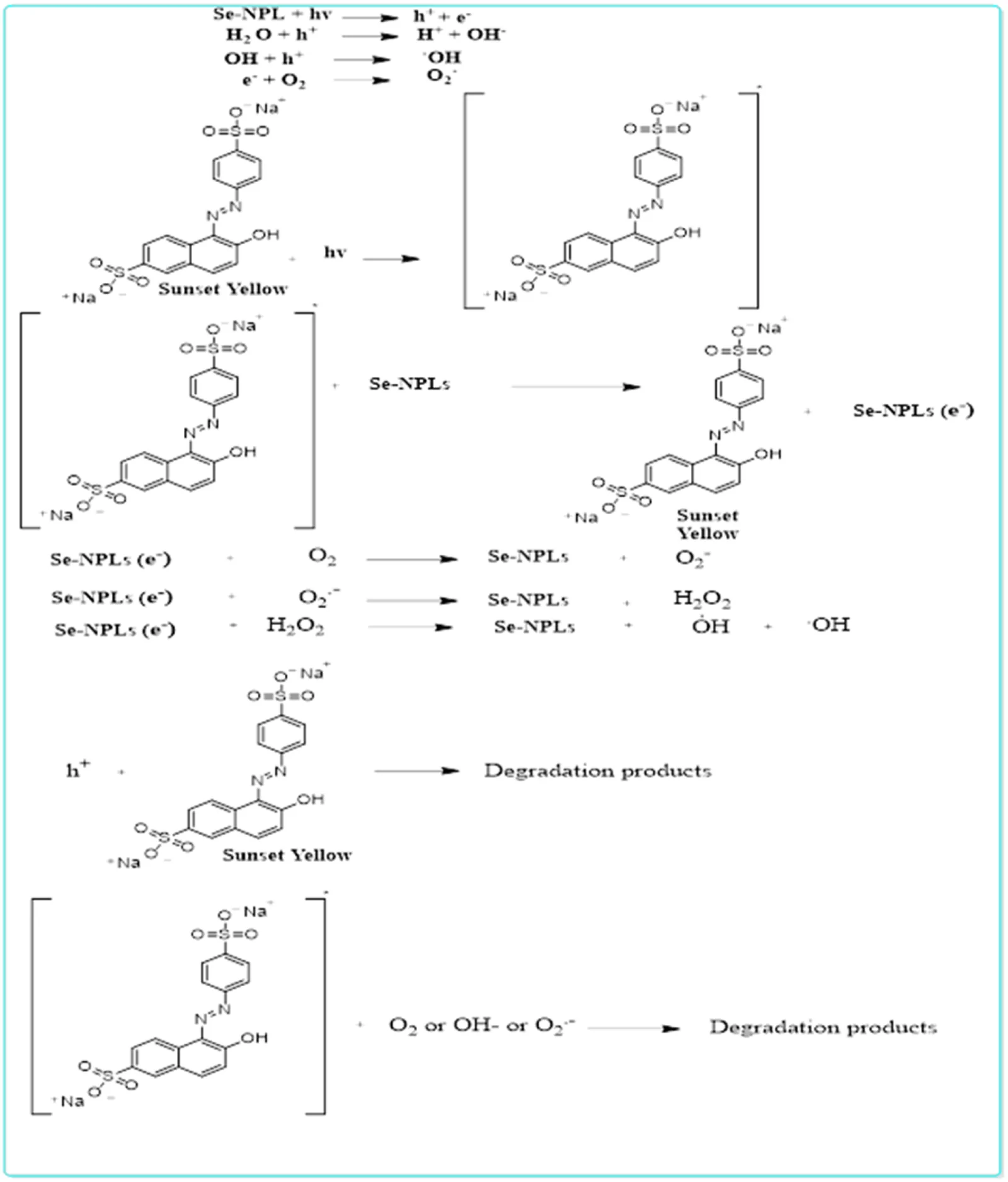
Schematic of the photocatalytic degradation mechanism of sunset yellow.^166^

The schematic illustrates the photocatalytic degradation mechanism of sunset yellow mediated by selenium nanoparticles (Se-NPLs) under light irradiation shown in Fig. 16.

Upon exposure to photons (*hν*), electron–hole pairs are generated, where photogenerated electrons (e^−^) react with dissolved oxygen to form superoxide radicals (O_2_˙^−^), which subsequently produce hydrogen peroxide (H_2_O_2_) and hydroxyl radicals (˙OH). Simultaneously, photogenerated holes (h^+^) oxidize water or hydroxide ions to generate additional ˙OH radicals. These highly reactive species initiate oxidative degradation of the sunset yellow molecule through successive attacks on the azo bond and aromatic rings, leading to structural breakdown and formation of intermediate products. Continuous oxidation results in fragmentation into smaller molecules, which are ultimately converted into non-toxic degradation products. This mechanism highlights the synergistic role of reactive oxygen species and charge carriers in driving efficient photocatalytic degradation of azo dyes.^167,168^

## 7. Mechanistic aspects of dye photodegradation over metal selenides

While Section 5 outlined the general thermodynamic framework of semiconductor photocatalysis, the efficiency of dye degradation over metal selenides is governed by mechanistic features specific to this class of materials. This section analyse multiple perspectives altogether to establish the structure–mechanism–performance relationships that underpin the rational design of selenide photocatalysts.

### 7.1 Visible-light absorption: the band-structure origin

**Fig. 17:**
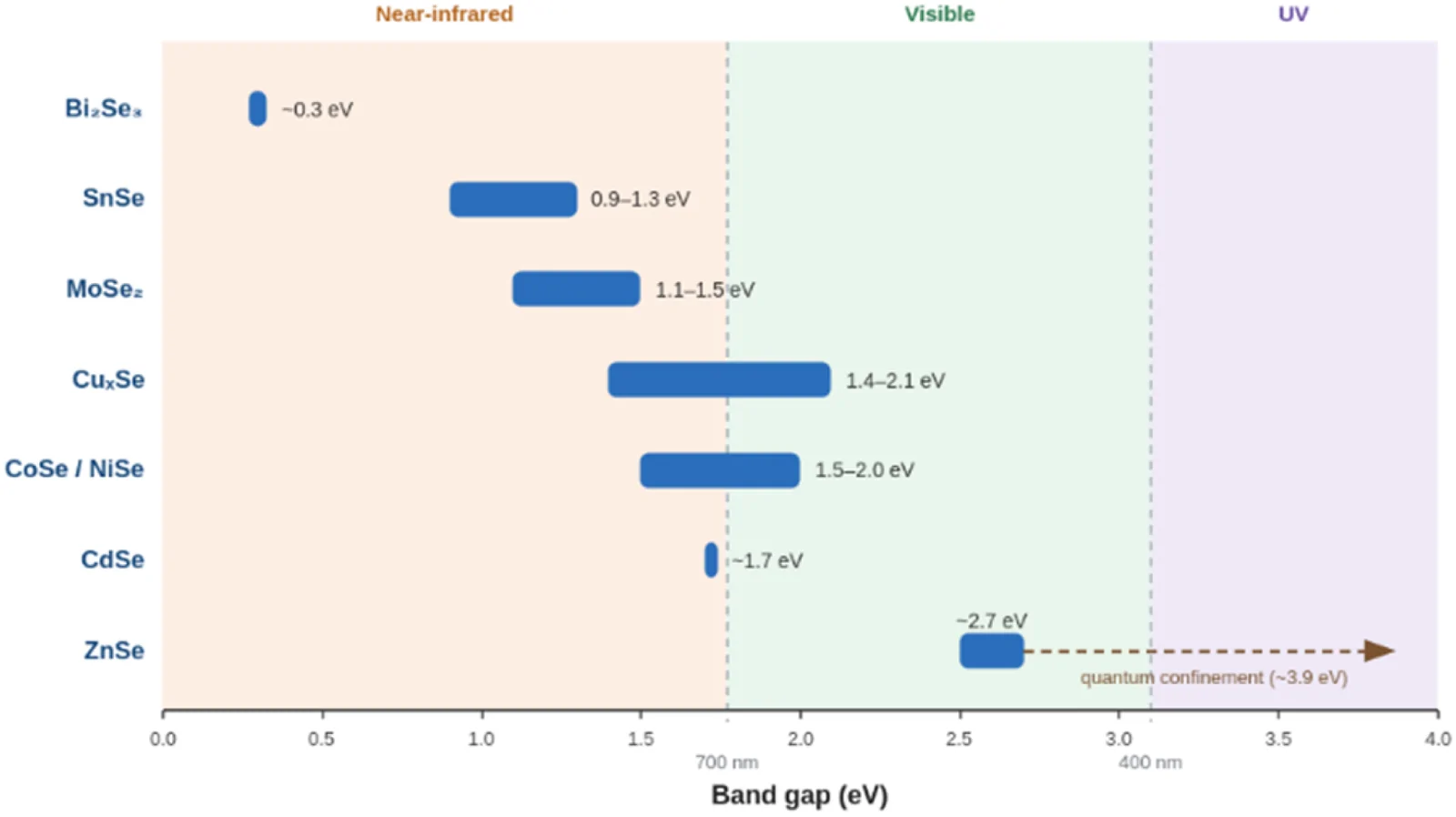
Band gaps of representative metal selenide photocatalysts plotted against the near-infrared, visible, and ultraviolet regions.

The defining mechanistic advantage of metal selenides originates in their electronic structure. In metal oxides, the valence band (VB) is dominated by low-lying O 2p orbitals, giving wide band gaps and UV-limited absorption. Substituting selenium raises the VB maximum through higher-energy Se 4p states, which hybridize with metal orbitals to narrow the band gap and shift the absorption onset into the visible and near-infrared.^169^ This is why selenides harvest a far larger fraction of the solar spectrum than the oxide analogues, and why compositional tuning (Section 3) translates so directly into tunable absorption. The is summarised value below in Fig. 17. The reported values span a wide, tunable range from 0.3 eV for Bi_2_Se_3_, through 0.9–1.3 eV (SnSe), 1.1–1.5 eV (MoSe_2_), 1.4–2.1 eV (Cu_*x*_Se), 1.5–2.0 eV (CoSe/NiSe), and 1.7 eV (CdSe), up to 2.7 eV for ZnSe, and can be widened to 3.9 eV by quantum confinement allowing the absorption onset to be positioned across the near-infrared, visible, and near-UV. Values are representative literature figures; ranges reflect differences in morphology, phase, and quantum confinement.

### 7.2 Charge separation and transport

The same narrow band gap that enables visible-light absorption also makes photogenerated electron–hole pairs prone to rapid recombination, which is the principal efficiency-limiting step. Selenides partially offset this through relatively high intrinsic carrier mobilities, but sustained activity requires the architectural strategies catalogued above, doping to introduce trap states, and, most effectively, heterojunction and carbon-mediated designs that physically separate carriers. The specific charge-transfer routes are described next.

### 7.3 Charge-transfer mechanisms in selenide heterostructures

#### 7.3.1 Type-II heterojunction

Two semiconductors with staggered band edges drive electrons to the lower conduction band (CB) and holes to the higher VB, achieving spatial separation. Charge separation improves, but the carriers accumulate on the weaker-reducing/oxidizing bands, so redox power is partly sacrificed. Selenide examples include MoSe_2_/ZnSe^96^ and ZnSe/CoSe.^94^

#### 7.3.2 Z-scheme

Geometrically similar to type-II, but the interfacial recombination of the weaker electrons and holes preserves the most-reducing CB electrons of one semiconductor and the most-oxidizing VB holes of the other retaining maximum redox power while still suppressing bulk recombination.

#### 7.3.3 S-scheme (step-scheme)

The modern refinement of the Z-scheme for two n-type semiconductors: the interfacial internal electric field and band bending selectively annihilate the low-energy carriers, leaving the strongly reducing and strongly oxidizing carriers intact. It is increasingly used to rationalize multicomponent selenide systems such as ZnO/ZnSe/MoSe_2_.^88^

#### 7.3.4 Schottky junction

At a semiconductor–metal contact, the Schottky barrier lets the metal act as an electron sink that blocks back-transfer and prolongs whole lifetime. This operates in noble-metal-decorated selenides such as Ag@CdSe^115^ and Ag–CdSe/GO,^129^ and in metallic–selenide contacts (*e.g.* PtSe_2_ (ref. 88)).

#### 7.3.5 p–n Junction

Pairing a p-type selenide (several copper selenides are intrinsically p-type) with an n-type semiconductor builds a space-charge region whose internal field separates carriers as soon as they form, before recombination competes.

#### 7.3.6 Carbon-mediated electron transfer

**Fig. 18:**
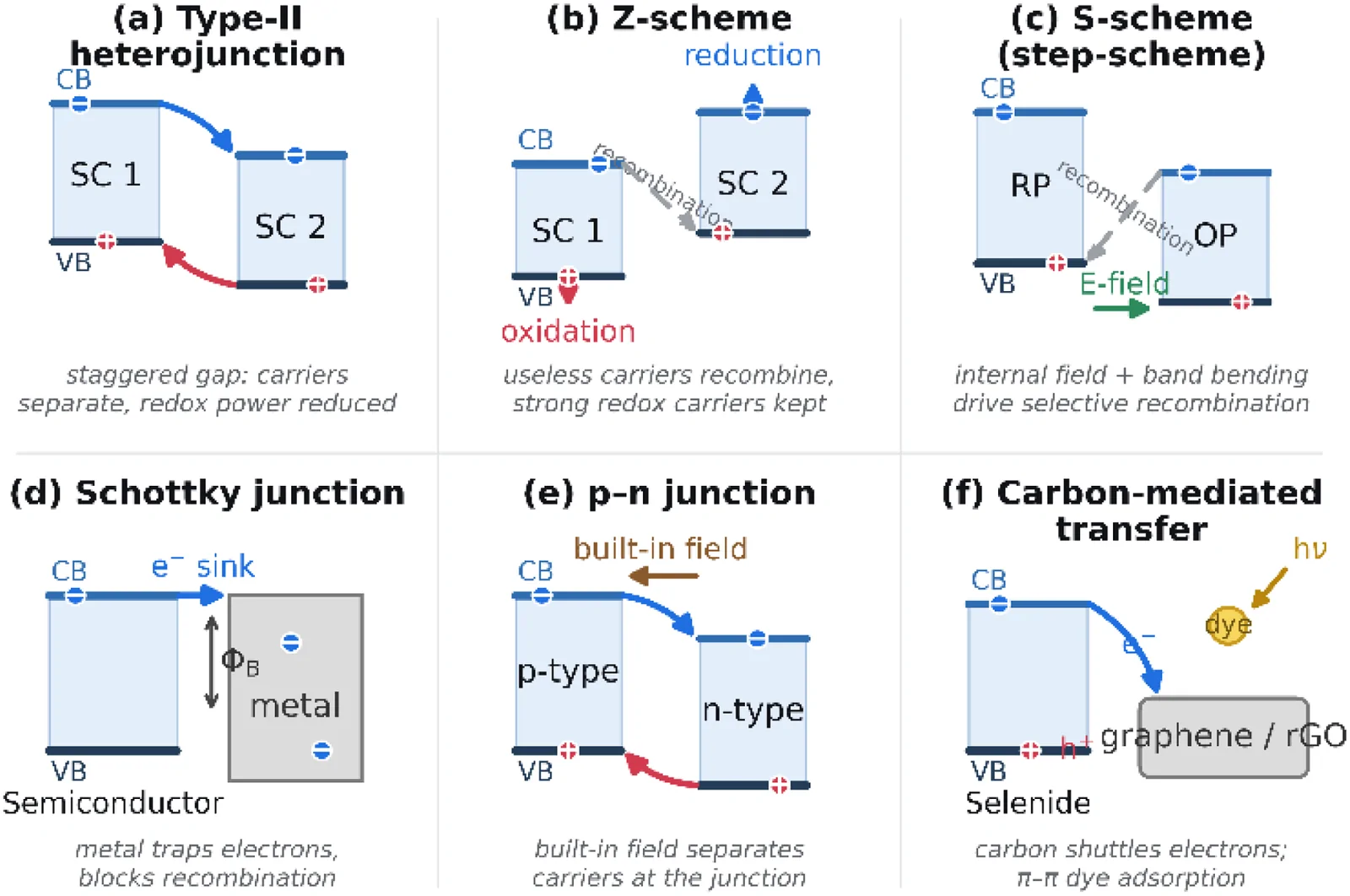
Principal interfacial charge-transfer mechanisms in metal selenide photocatalysts: (a) type-II heterojunction, (b) Z-scheme, (c) S-scheme, (d) Schottky junction, (e) p–n junction, and (f) carbon-mediated electron transfer. CB, conduction band; VB, valence band; e^−^, photogenerated electron; h^+^, hole.

Graphene, rGO, and CNTs are not band-alignment partners but conductive mediators: they accept and shuttle CB electrons away from the selenide (suppressing recombination) while their π-conjugated surface adsorbs dye *via* π–π stacking, pre-concentrating substrate at the reaction zone. This underlies rGO/SnSe.^90^ CdSe/rGO^98^ and graphene–PtSe_2_.^87^ These activities are summarised in Fig. 18.

### 7.4 ROS generation and interfacial redox

**Fig. 19:**
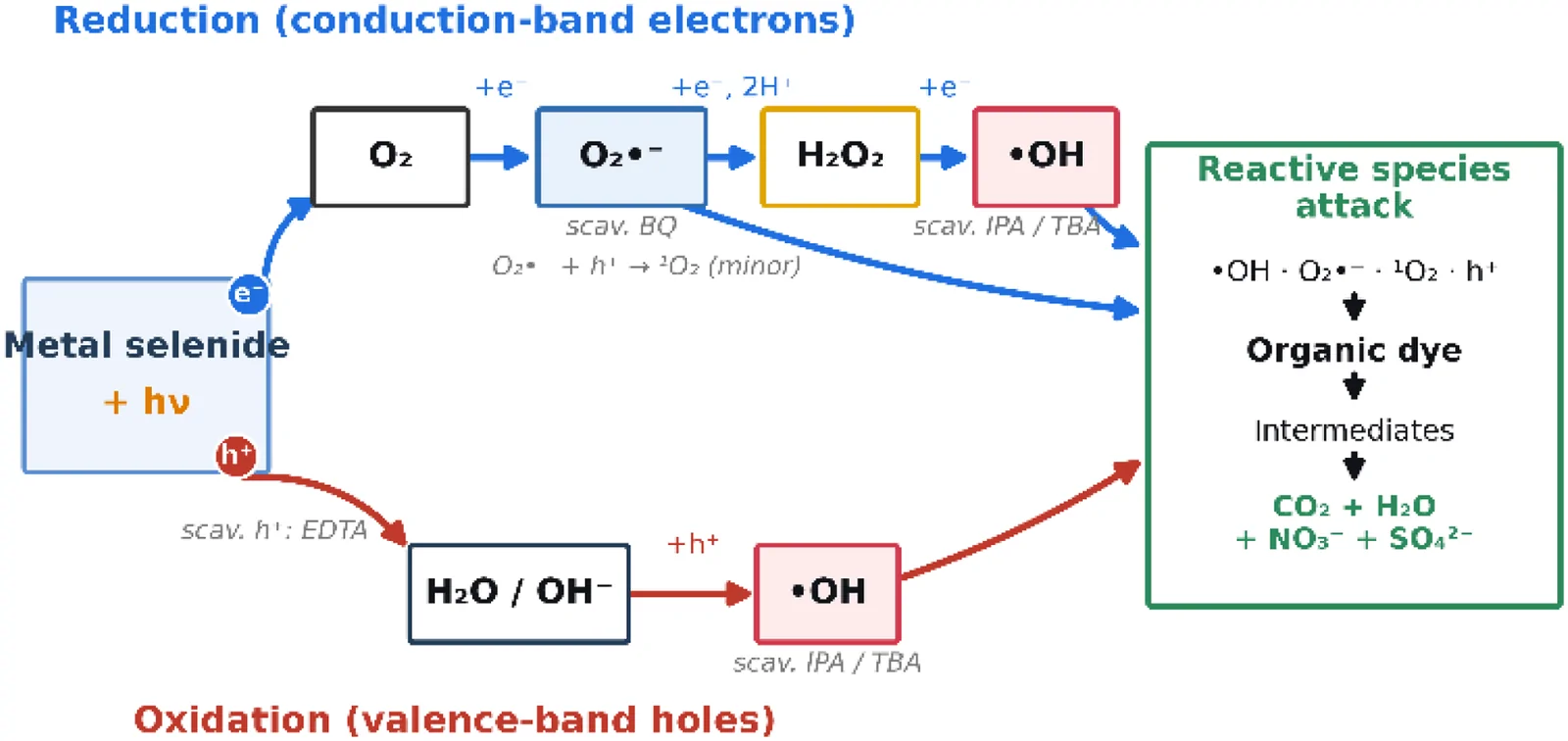
Reactive-oxygen-species generation network over metal selenide photocatalysts. Conduction-band electrons drive the reductive cascade O_2_ → O_2_˙^−^ → H_2_O_2_ → ˙OH, while valence-band holes oxidize H_2_O/OH^−^ to ˙OH; the resulting species (˙OH, O_2_˙^−^, ^1^O_2_, h^+^) attack the dye, yielding intermediates that mineralize to CO_2_, H_2_O, and inorganic ions. Common radical scavengers are shown in grey (BQ, benzoquinone; IPA, isopropanol; TBA, *tert*-butanol; EDTA).

Whether a given selenide can generate a particular reactive oxygen species is dictated by its band-edge positions relative to the relevant redox potentials. Reduction of O_2_ to superoxide (O_2_/O_2_˙^−^ ≈ −0.33 V *vs.* NHE) requires a CB more negative than this value, whereas oxidation of water/hydroxide to hydroxyl radical (˙OH/OH^−^ ≈ +1.99 V *vs.* NHE) requires a VB more positive. Because the Se 4p-derived VB of many selenides is relatively high (less positive), a substantial number of selenide photocatalysts can readily reduce O_2_ to O_2_˙^−^ but cannot directly oxidize water to ˙OH. In these systems, superoxide is typically the dominant primary ROS, and hydroxyl radicals form indirectly *via* O_2_˙^−^ → H_2_O_2_ → ˙OH rather than by direct hole oxidation. This thermodynamic picture explains why radical-scavenger studies on selenides so often identify O_2_˙^−^ (and h^+^) as the principal active species, and it provides a rational criterion raising the VB-hole oxidizing power through heterojunction or Z-/S-scheme design for enabling direct ˙OH pathways. The dominant reactive species reported across representative selenide systems are summarized in Table 11, together with the scavenger and spectroscopic evidence on which each assignment rests. These activities are summarised in Fig. 19.

**Table 11:** Dominant reactive oxygen species identified in representative metal selenide photocatalytic systems, with the corresponding scavenger and ESR/EPR evidence and proposed degradation pathways

Photocatalyst	Dye	Scavengers tested	Dominant ROS	ESR/EPR evidence	Proposed pathway	Ref.
ZnO/ZnSe/MoSe_2_ (ternary)	Methyl orange	BQ (O_2_˙^−^), *t*-BuOH (˙OH), ammonium oxalate (h^+^)	O_2_˙^−^, ˙OH and h^+^ (all three contribute; O_2_˙^−^ dominant in the binary ZnO/ZnSe)	DMPO–O_2_˙^−^ and DMPO–˙OH signals detected under visible light, absent in the dark		88
PANI–Bi_2_Se_3_ (PBS25)	Methyl orange, rhodamine B, malachite green	*p*-Benzoquinone (O_2_˙^−^), TBA (˙OH), sodium oxalate (h^+^), K_2_S_2_O_8_ (e^−^), EDTA	O_2_˙^−^ (dominant), with h^+^ and ˙OH contributing	Not reported (radical-trapping only)	CB electrons of PANI/Bi_2_Se_3_ reduce O_2_ → O_2_˙^−^; radicals + holes cleave the dye; dye-sensitization also contributes → mineralization	93
Ni-doped Bi_2_Se_3_ (+H_2_O_2_)	Malachite green	*p*-Benzoquinone (O_2_˙^−^), TBA (˙OH), sodium oxalate (h^+^), K_2_S_2_O_8_ (e^−^)	O_2_˙^−^ prominent; e^−^, h^+^ and ˙OH all contribute	Not reported (radical-trapping only)	CB electrons reduce O_2_ → O_2_˙^−^ → H_2_O_2_ → ˙OH; radicals degrade MG → mineralization	83

Consistent with the band-position analysis above, superoxide (O_2_˙^−^) and photogenerated holes are the species most frequently identified as dominant, whereas direct hydroxyl–radical pathways are reported mainly for heterojunction and Z-/S-scheme systems in which strongly oxidizing holes are retained. This convergence between the thermodynamic prediction and the experimental scavenger evidence reinforces O_2_˙^−^-mediated oxidation as the characteristic degradation route for narrow-gap selenide photocatalysts.

### 7.5 Dye adsorption

**Fig. 20:**
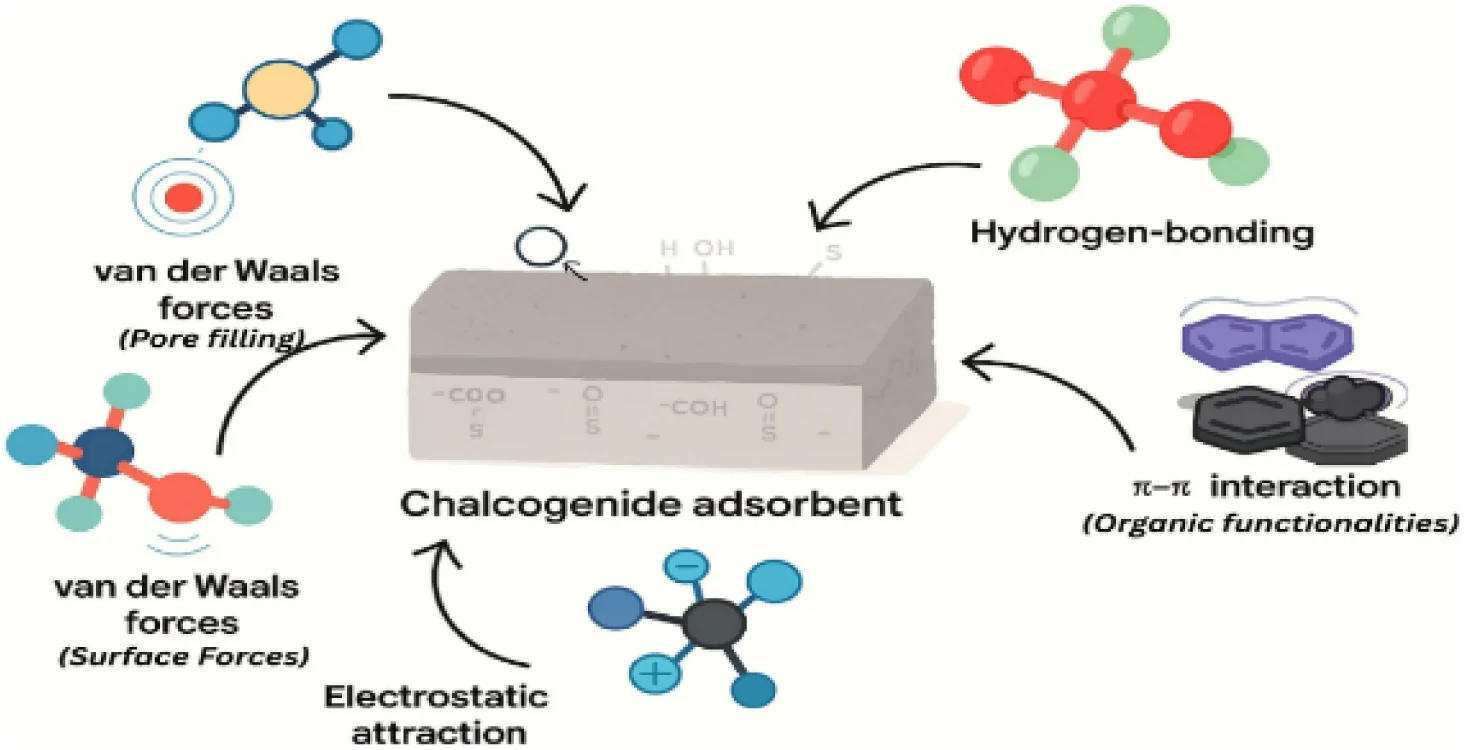
Surface forces governing the adsorption of organic contaminants on chalcogenide-based photocatalysts: electrostatic attraction, van der Waals forces, hydrogen bonding, and π–π interactions. Reproduced from Alfred, M. O. *et al.*, ‘Chalcogenides: recent advances in their environmental applications’, *Energy, Ecology and Environment* (2025), DOI: https://www.doi.org/10.1007/s40974-025-00389-1. Licensed under CC BY 4.0.^170^

Efficient degradation begins with adsorption: dye must reach the catalyst surface before interfacial charge transfer can occur. Adsorption on selenide systems is governed by electrostatic complementarity between the (pH-dependent) surface charge and the ionic character of the dye favouring cationic dyes on negatively charged surfaces and *vice versa* and, in composite systems, by π–π stacking on carbon supports and by the abundant functional groups of chitosan and cellulose matrices. Strong pre-adsorption both concentrates substrate at active sites and, in visible-light tests, raises the caveat of dye-sensitized self-degradation discussed in Section 7.6. These adsorption interactions are summarized in Fig. 20.

### 7.6 Dye-sensitization and its influence on apparent activity

A methodological caveat applies to almost all visible-light dye-degradation data, including much of the literature surveyed above. Coloured dyes are themselves strong visible-light absorbers, such as methylene blue (*λ*_max ≈ 663 nm), rhodamine B (≈553 nm), crystal violet (≈590 nm), together with malachite green, methyl orange, and Congo red and efficiently harvest visible photons. On excitation, an adsorbed dye molecule (Dye → Dye*) can inject an electron into the conduction band of the semiconductor, initiating the same radical cascade (O_2_ → O_2_˙^−^ → …) that drives photocatalysis, but with the dye, not the catalyst, acting as the light-harvesting antenna. This dye-sensitized pathway degrades the dye even when the semiconductor absorbs little visible light and therefore inflates the apparent visible-light activity of the catalyst.

The consequence is that a decolorization measurement made under visible light with a coloured dye as the sole probe cannot, by itself, distinguish true semiconductor-driven photocatalysis from self-sensitized dye degradation. The artefact is most severe for wide-band-gap components for example ZnSe, and the ZnO or TiO_2_ partners used in several of the composites surveyed here which absorb weakly in the visible and thus benefit most from sensitization. Intrinsically narrow-gap selenides are genuinely visible-active and correspondingly less susceptible, but a sensitization contribution can rarely be excluded outright.

Rigorous discrimination requires control experiments that few of the surveyed studies report: (i) degradation of a colourless substrate (*e.g.* phenol) under identical conditions; (ii) comparison of activity under filtered visible *versus* UV irradiation, or under monochromatic light outside the dye's absorption band; (iii) wavelength-dependent (action-spectrum) measurements; and (iv) mineralization assays (TOC/COD), since sensitized decolorization frequently reflects chromophore cleavage rather than true degradation (Section 6.6-linked, see decolorization-*vs.*-mineralization discussion). Reporting apparent rate constants alongside such controls would substantially improve the reliability of performance claims across this field.

## 8. Parameters affecting photocatalytic degradation

The efficiency and rate of photocatalytic dye degradation are determined by intrinsic factors (dye and photocatalyst properties) and extrinsic factors (dye concentration, pH, temperature, light intensity, and nanomaterial characteristics).^171^ The efficiency of photocatalytic dye degradation depends on parameters such as pH, dye concentration, catalyst size and dosage, temperature, light intensity, and electron acceptors.^167^

### 8.1 Effect of pH

The pH of the solution is a critical operational parameter in wastewater treatment, as it significantly influences the efficiency of photocatalytic degradation processes.^172^ Solution pH significantly influences photocatalytic dye degradation. In acidic media, photogenerated holes (h^+^) dominate the oxidation process, whereas at neutral to alkaline pH, hydroxyl radicals (˙OH) become the main oxidizing species. Alkaline conditions enhance ˙OH radical formation on the catalyst surface, improving degradation efficiency.^173^ Ali *et al.* reported that dye degradation increased as pH rose from 3 to 7 but declined above pH 8. Excess hydroxyl ions (OH^−^) reduce attraction to the anionic photocatalyst surface, lowering degradation efficiency.^174^ Demir *et al.* demonstrated that the degradation efficiency of both RhB and MB increased with rising pH when using ZnO and Sn/ZnO photocatalyst samples.^175^ Shehzad *et al.* reported that dye degradation is fastest in neutral solution (*k* = 0.09 min^−1^), higher than in acidic (0.08 min^−1^) or basic (0.07 min^−1^) media. TiO_2_ surface charge modulates MB adsorption and reaction rate, with excess H^+^ reducing efficiency.^176^

### 8.2 Effect of oxidizing agent

Inorganic oxidants (O_3_, H_2_O_2_, BrO_3_^−^, S_2_O_8_^2−^, ClO_4_^−^) enhance TiO_2_/UV photocatalysis by trapping electrons, inhibiting recombination, and generating additional oxidizing radicals that improve dye degradation.^167^ Ullah *et al.* reported that RBV-5R degradation reached 99% at 6 mM H_2_O_2_ and pH 10. H_2_O_2_ enhanced ˙OH radical formation, maximizing degradation, but higher concentrations led to radical recombination and reduced efficiency.^177^ Andronic *et al.* reported that H_2_O_2_ enhances dye degradation by acting as an electron scavenger, inhibiting electron–hole recombination on the semiconductor surface. Its efficiency depends on proper dosage, pollutant type, and concentration.^178^ Sivakumar *et al.* demonstrated that adding 6 mM H_2_O_2_ enhanced RY 84 and RB 5 degradation to 100% by increasing ˙OH radical formation and suppressing e^−^/h^+^ recombination, higher dosages offered no further improvement.^179^

### 8.3 Effect of catalyst dosage

Catalyst loading is a crucial factor influencing photocatalytic degradation efficiency.^174^ Varoius studies have shown that the amount of photocatalyst strongly affects the degradation of organic pollutants during photocatalysis.^180^ Vu *et al.* showed that increasing Au/ZnO catalyst loading enhances active sites and ˙OH/O_2_˙^−^ radical formation, boosting photocatalytic activity, but excessive loading causes light scattering and limits light penetration.^181^ Gupta *et al.* found that increasing the photocatalyst dose from 0.05 to 0.50 g L^−1^ enhanced dye degradation by providing more active sites, with 0.50 g L^−1^ yielding maximum efficiency for subsequent experiments.^182^ Abuzeyad *et al.* reported that increasing the photocatalyst dose from 5 to 15 mg improved methylene blue degradation, reaching 94% at 15 mg due to greater availability of active sites.^183^

### 8.4 Effect of concentration of dye

Initial dye concentration is a crucial parameter in photocatalytic systems, as it governs degradation kinetics by influencing the interaction between hydroxyl radicals (˙OH) and the catalyst's active sites.^184^ According to Ali, N. *et al.* Increasing dye concentration reduces photocatalytic degradation efficiency, as enhanced light absorption by dye molecules limits photon penetration and restricts their diffusion to the catalyst surface, suppressing the reaction.^185^ Ahmad *et al.* found that crystal violet degradation was optimal at 30 ppm, where dye adsorption on catalyst sites was maximized. Higher concentrations reduced efficiency due to surface saturation and limited light penetration.^101^ Kansal *et al.* reported that increasing RB5 and RO4 concentrations decreased photocatalytic decolorization rates due to reduced photon penetration. Complete decolorization occurred rapidly at low concentrations (10–25 mg L^−1^) but required longer irradiation at higher concentrations (50 mg L^−1^).^186^ Chen *et al.* found that increasing MO, CR, and DB38 concentrations (10–50 mg L^−1^) reduced photodegradation after 10 min due to active-site saturation, limited O_2_/˙OH adsorption, and hindered photon penetration.^187^ Shathy, R. A., *et al.* demonstrated that increasing the initial dye concentration can significantly suppress photocatalytic efficiency. At elevated concentrations, the quantity of generated reactive species becomes inadequate compared to the excess dye molecules, limiting effective degradation. Furthermore, higher dye loading reduces light transmission through the solution, decreasing photon availability at the catalyst surface. The formation and adsorption of intermediate by-products also obstruct active sites, hinder reactive radical formation, and consequently impair overall photocatalytic performance.^188^ Kumar, A. P., *et al.* reported that increasing dye concentration led to a decrease in degradation efficiency for both Pd-γ-Al_2_O_3_ and PdO-γ-Al_2_O_3_ photocatalysts. Higher concentrations result in greater adsorption of dye molecules on the catalyst surface, which limits photon absorption and reduces the generation of reactive hydroxyl (˙OH) radicals. Consequently, active sites are partially blocked, lowering photocatalytic efficiency. Maximum degradation was observed at 5 ppm for BCG and BTB, and at 10 ppm for MO and MB, with efficiency declining at higher concentrations.^189^

### 8.5 Effect of irradiation time

Reaction time is a key factor affecting photocatalyst performance, an ideal photocatalytic process should achieve rapid and efficient pollutant degradations.^190^ Nawaz *et al.* observed that methylene blue degradation under solar irradiation reached 92% after 150 min, after which active-site saturation and dye–dye repulsion limited further removal.^191^ Rahman *et al.* reported that dye degradation increased over 120 min, with NZVI@BiFeO_3_/g-C_3_N_4_ achieving 97% removal, longer reaction times did not improve degradation.^192^ Rahman *et al.* demonstrated that ZnO NPs under UV light degraded 95% of RhB in 70 min, with most degradation occurring in the first 30 min, no activity was observed in the dark.^193^ Salama, A., *et al.*, reported that during the initial 90 minutes of irradiation. The degradation rate was rapid, achieving 80% for IC and 52% for MB. Thereafter, the rate gradually slowed, reaching complete degradation at 180 minutes for IC and 300 minutes for MB. The initial high degradation is attributed to the abundant amino-functionalized TiO_2_ nanoparticles on the CA–CNT composite nanofiber surface, while the subsequent decrease results from progressive catalyst consumption.^194^

## 9. Practical challenges and environmental considerations

The preceding sections establish that metal selenides are efficient and mechanistically versatile dye photocatalysts. Their translation from laboratory demonstrations to practical wastewater treatment, however, is constrained by three issues that the current literature addresses only partially: the distinction between decolorization and genuine mineralization, the environmental risk posed by the catalysts themselves, and performance under realistic effluent conditions.

### 9.1 Decolorization, degradation, and mineralization

A recurring limitation of the studies surveyed here is that performance is almost always quantified as decolorization loss of the dye's visible absorbance which is not equivalent to degradation, mineralization, or detoxification. Decolorization reflects only cleavage of the chromophore (*e.g.* the azo or conjugated system); the resulting colourless intermediates, such as the aromatic amines released from azo-dye cleavage, are frequently more toxic and more persistent than the parent dye. True degradation requires fragmentation of these intermediates, and mineralization their complete conversion to CO_2_, H_2_O, and inorganic ions is confirmed only by total-organic-carbon (TOC) or chemical-oxygen-demand (COD) removal, not by absorbance. Very few of the selenide studies reviewed report TOC/COD data, and fewer still assess residual toxicity. High reported “degradation” efficiencies should therefore be interpreted with caution: a system achieving 99% decolorization may achieve substantially lower mineralization. Systematic reporting of TOC/COD removal and toxicity endpoints alongside decolorization is essential for meaningful evaluation.

### 9.2 Catalyst toxicity, leaching, and environmental risk

A consideration frequently overlooked when claiming practical applicability is the environmental risk posed by the photocatalysts themselves. Several of the most active selenides incorporate toxic constituents' cadmium (CdSe), selenium, and other heavy metals (Bi, Cu, Ni, Co, Sn) whose release during operation would constitute secondary contamination. Selenium and metal-ion leaching can occur under illumination through photocorrosion, to which chalcogenides are particularly prone, and the leached species are themselves hazardous; selenium in particular has a narrow margin between essential and toxic concentrations. Robust evaluation therefore requires measurement of dissolved metal/selenium after each cycle (*e.g.* by ICP-MS), assessment of the photostability and structural integrity of the recovered catalyst, and consideration of the toxicity and safe disposal of the spent material. Immobilizing selenides on chitosan, cellulose, or polymer supports as in several composites surveyed here mitigates leaching and aids recovery but does not eliminate the concern. Claims of environmental benignity should be substantiated by leaching and stability data, and the life-cycle impact of the catalyst weighed against the pollutant it removes.

### 9.3 Performance under real wastewater conditions

Nearly all the performance data reviewed were obtained with single-dye synthetic solutions in deionized water under idealized laboratory illumination conditions far removed from real textile effluent. Actual wastewater contains co-existing inorganic ions (chloride, sulfate, carbonate, phosphate) that can scavenge reactive radicals or compete for active sites; natural organic matter and surfactants that compete for light and oxidant; and high, variable salinity, turbidity, and pH, together with mixtures of dyes and auxiliaries rather than a single chromophore. Turbidity and colour further attenuate light penetration, lowering photon availability. Moreover, the powder catalysts used in most studies require post-treatment separation that is impractical at scale; immobilization, magnetic recovery, and continuous-flow or fixed-bed configurations are needed for deployment, yet only a handful of the surveyed studies such as the magnetically fixed-bed Fe_3_O_4_/ZnO/ZnSe system^97^ address these. Validation against genuine textile effluents, with long-term stability testing over extended operation, is a prerequisite for translating these laboratory results into viable technologies.

## 10. Conclusions

This review provides a comprehensive and critical evaluation of metal selenide-based photocatalysts for the degradation of organic dyes, underscoring their growing significance in advanced wastewater treatment technologies. The collective body of literature clearly demonstrates that metal selenides possess distinct advantages, including narrow band gaps, strong visible-light absorption, and favourable electronic structures, which render them highly effective for photocatalytic applications under solar irradiation. These intrinsic properties enable efficient generation and utilization of photogenerated charge carriers, positioning metal selenides as promising alternatives to conventional wide band gap semiconductors. A detailed analysis reveals that while pristine metal selenides exhibit moderate photocatalytic performance, their efficiency can be substantially enhanced through rational structural and compositional engineering. Strategies such as heterojunction formation, incorporation of co-catalysts, and development of ternary or hybrid systems significantly improve charge carrier separation and prolong the lifetime of photogenerated electrons and holes. This, in turn, promotes the generation of reactive oxygen species (˙OH and O_2_˙^−^), which are primarily responsible for the oxidative degradation of dye molecules *via* pathways involving azo bond cleavage, dealkylation, hydroxylation, and eventual mineralization into environmentally benign products.

Moreover, the review highlights the critical role of synthesis methodologies such as hydrothermal, solvothermal, and co-precipitation techniques in tailoring the physicochemical properties of metal selenides, including particle size, morphology, crystallinity, and surface area. These parameters directly influence light absorption, surface reactivity, and overall photocatalytic efficiency. In parallel, operational conditions such as solution pH, catalyst dosage, initial dye concentration, and light source are identified as key governing factors that significantly affect degradation performance and reaction kinetics across different systems. Despite these advancements, several challenges remain, including catalyst stability, recyclability, large-scale implementation, and performance under real wastewater conditions. Addressing these limitations requires the development of robust, scalable, and environmentally benign photocatalytic systems with enhanced durability and efficiency under natural sunlight.

In conclusion, metal selenide-based photocatalysts represent a highly promising class of materials for next-generation, eco-friendly wastewater treatment. Continued efforts in material design, mechanistic understanding, and process optimization are expected to further advance their practical applicability, paving the way toward sustainable and efficient environmental remediation technologies.

## 11. Future recommendations

Future research should prioritize the rational design of next-generation metal selenide photocatalysts with enhanced efficiency, selectivity, and long-term stability for practical wastewater remediation. In particular, the development of ultrathin two-dimensional (2D) and quantum-confined metal selenides offers a promising pathway to maximize surface-active sites, tailor band structures, and accelerate charge carrier dynamics. The integration of artificial intelligence (AI)-guided material design is expected to further revolutionize this field by enabling predictive optimization of composition, morphology, and adaptive structural transformations for highly selective and energy-efficient dye degradation. Concurrently, emphasis should be placed on engineering multifunctional systems, including solar-driven, self-healing, and dual-stimuli-responsive photocatalysts, capable of operating under real environmental conditions.

Advancements in material architecture, such as self-assembling hierarchical nanostructures and engineered heterointerfaces, should be coupled with photon up-conversion strategies to extend light absorption across the solar spectrum and enhance photocatalytic performance. Moreover, integrating metal selenide photocatalysts with complementary technologies such as membrane filtration, photoelectrochemical hydrogen production, and hybrid biotic–abiotic systems can enable continuous, synergistic, and sustainable wastewater treatment processes. The incorporation of *in situ*, real-time monitoring techniques will further facilitate mechanistic understanding, process control, and adaptive response to dynamic environmental conditions.

In addition, future efforts must address critical challenges related to catalyst durability, recyclability, and scalability, particularly under complex real wastewater matrices containing competing ions and organic species. Life-cycle assessment, green synthesis approaches, and environmental risk evaluation should be systematically incorporated to ensure safe and sustainable deployment. Collectively, these strategies will pave the way for the development of intelligent, high-performance metal selenide-based photocatalytic systems, advancing their transition from laboratory research to large-scale, real-world environmental applications.

## Author contributions

Nawaz Khan: conceptualization, methodology, investigation and original draft. Waqar Ahmad: visualization, data curation, writing – original draft. Muhammad Khan: editing, writing, review. Adnan Khan: supervision, conceptualization writing – original draft, visualization. Mahnoor Amjad: statistical analysis and modelling. Sabir Khan: investigate and supervised specific finding of the work. Nauman Ali: verified analytical methods. Nisar Ali: writing – review and editing. Jiaojiao Zheng: supervision, methodology, review and editing.

## Conflicts of interest

The authors declare no conflicts of interest.

## Supplementary Material

RA-016-D6RA04616A-s001

## Data Availability

No new data were created or analysed in this work. As this article is a review, all information discussed is derived from previously published literature, which has been appropriately cited within the manuscript. Supplementary information (SI): the complete comparison tables of metal selenide-based photocatalysts for the degradation of the organic dyes discussed in the main text, listing for each system the full set of experimental parameters including initial dye concentration, catalyst dosage, solution pH, specific surface area, dominant reactive oxygen species (ROS), irradiation time, and reusability that are presented in condensed form in the corresponding main-text tables. See DOI: https://doi.org/10.1039/d6ra04616a.
