# Supplementary material for: Transition metal selenide-based photocatalysts for the degradation of organic dyes: materials design, mechanistic insights, and practical applicability
Source: RSC Adv. 2026 Aug 14;16(40):45428–55. doi: 10.1039/d6ra04616a (PMC13474994; doi:10.1039/d6ra04616a)
Supplement: Supplementary file 1 [file RA-016-D6RA04616A-s001.pdf]

Supplementary Information (SI) for RSC Advances.
This journal is © The Royal Society of Chemistry 2026

Transition Metal Selenide-Based Photocatalysts for the Degradation of Organic Dyes: Materials Design, Mechanistic Insights, and Practical Applicability

Nawaz Khan [a], Waqar Ahmad [a], Muhammad Nauman Ahmad Khan [b*], Mahnoor Amjid [a], Adnan Khan [a,c], Sabir Khan [d], Nisar Ali [e], Nauman Ali [a], and Jiaojiao Zheng [*b]

a. Institute of Chemical Sciences, University of Peshawar, Khyber Pakhtunkhwa 25120, Pakistan.

b. Energy & Bioproducts Research Institute (EBRI), College of Engineering and Physical Sciences (EPS), Aston University, Aston Triangle, Birmingham, B4 7ET, United Kingdom.

c. Center of Chemical, Pharmaceutical and Food Sciences, Federal University of Pelotas, Pelotas, 96010-900, RS, Brazil.

d. Materials Science and Engineering (PPGCEM), Technological Development Center, Federal, University of Pelotas (UFPel), RS, 96010-610, Pelotas, Brazil.

e. Department of Basic and Applied Sciences, College of Applied and Health Sciences, A'Sharqiyah University, P.O. Box 42, Ibra P.O. 400, Sultanate of Oman.

* Corresponding author: Jiaojiao Zheng, j.zheng2@aston.ac.uk.

Table 1 Overview of Alizarin red degradation by metal selenide based photocatalysts.

Photocatalyst	Synthesis Routes	Dye and concentration	Catalyst Dose	pH	Irradiation time (min) and source	Removal (%)	Eg (eV)	kapp (min^{-1})	Reusability	References
MnNiSeCs-NPs	co-precipitation	AR and TZ, 30 ppm	AR (0.1 g); TZ (2 g)	7	75, sunlight	ARS (98)；TZ (96)	/	AR (0.054); TZ (0.049)	4 runs: slight decrease.	[1]
TMS-CM	solvothermal	ARS, 0.15 mM	0.5g	4	180, sunlight	95.4	1.8	0.061	6th run: 86.7%.	[2]

Table 2 Overview of Congo red degradation by metal selenide based photocatalysts.

Photocatalyst	Synthesis route	Dyes and concentrations	Catalyst Dosage	pH	Irradiation time (min) and sources	Removal (%)	Eg (eV)	kapp (min^{-1})	Reusability	References
ZBiSe-CM	Solvothermal	CR, 40 ppm	0.225 g	8	120, solar light	99.63	2.35	0.100	5th run: 98.80%.	[3]
FeNiSe-CHM	Solvothermal	CR, 60 ppm	0.20	6	140, sunlight	99	2.09	0.043	5 runs: slight decrease	[4]
$AgInSbSe_3$	Hydrothermal	CR, 10 ppm	0.02g	/	75, sunlight	77.8	1.97	0.018	3 runs: 77.8 →75%	[5]
BCSN-CM	Solvothermal	CR, 90 ppm	0.60	8	100, sunlight	85	2.48	0.015	5 runs: slight decrease	[6]
Bi_2Se_3	Solvothermal	CR, RB	/	/	90, visible light	CR (35), RB (90)	1.20	CR (0.032), RB (0.0365)	5 runs: slight decrease	[7]
Ni/Bi_2Se_3	Solvothermal	CR, RB	/	/	90, visible light	CR (94), RB (97)				
Quaternary metal selenide	Hydrothermal process	CR	/	/	60, sunlight	86.9%	2.01	/	/	[8]

Table 3 Overview of Crystal violet degradation by metal selenide based photocatalysts

Photocatalyst	Synthesis route	Dyes and concentrations	Catalyst dosage	pH	Irradiation time (min) and source	% degradation	Eg (eV)	kapp (min^{-1})	Reusability	References
BISe-CM	Solvothermal	CV, 30 ppm	0.2 g	8	150, 99.04	99.04	2.68	0.01 min^{-1}	4 runs: 92.58%	[9]
CS-PANI	Chemical precipitation	CV, 40 ppm	0.5g	8	80, 89.5	89.5	2.74	/	6 runs: 71.4%	[10]

Table 4 Overview of Malachite green degradation by metal selenide based photocatalysts.

Photocatalyst	Synthesis route	Dye and concentration	Catalyst dosage	Irradiation time and source	% degradation	kapp (min^{-1})	Reusability/ stability	References
Ni/Bi_2Se_3	Solvothermal	MG	/	5 min, visible light	100	/	/	[11]
CdSe@CA, Ag-CdSe@CA, CdSe/GO@CA, Ag-CdSe/GO@CA	Hydrothermal	MG, 120mg/mL,	5ppm	25 min	92, 94, 94, 97	3.17, 3.51, 3.45, 4.17	5 cycles: slight decrease	[12]
rGO/SnSe	Hydrothermal	MG	/	Visible light	95.52 83.52	/	Excellent	[13]
SeNPs	Eco-friendly method	MG	1mg/L	240 min, sunlight	89	/	Highly stable	[14]

Table 5 Overview of Methylene blue degradation by metal selenide based photocatalysts.

**Photocatalyst**	**Synthesis route**	**Dyes and concentrations**	**Catalyst dose**	**pH**	**S_{BET} (m^2 g^{-1})**	**Irradiation time and source**	**Removal (%)**	**Eg (eV)**	**k_{app} (min^{-1})**	**Reusability/Stability**	**References**
CdSe and ZnSe nanoparticles	co-precipitation and hydrothermal	MB	/	/	/	0-150 min, UV light	78	CdSe~2.1; ZnSe~2.6	/	/	[15]
Ag@CdSe/Zeolite	Facile synthesis	MB, 7.17 mg/L	0.03g/50 mL	8	/	40 min, visible light	89.98	/		/	[16]
ZnSe/graphene Nanocomposites	hydrothermal method	MB, 35 mg/L	10 mg.	4	/	360, visible light	99.6	/	0.212	/	[17]
$Cu_{1.8}Se$ NPs	co-precipitation	MB, 1 g/mol	0.05 g,	/	/	0–60 min, incandescent bulb	Maximum	/	/	/	[18]

						light					
CdSe	thermal treatment	MB, 20 mg/L	0.02 g/50 mL	8	26.71	20, visible light	Predicted: 92.80 Experimental: 90.87	2.55	0.038	10 runs: decreased by 31.11%	[19]
$Cu_{2-X}Se$ NPs	co-precipitation	MB, 1 g/100 mL	0.05 g	/	/	120 min, solar light	90.30	2.1	0.0075	4 runs: slight decrease	[20]
$Cu_xSn_{1-x}Se$ nanocrystals	sonochemical exfoliation technique	MB, CV, 20 mL of 20ppm	20 mg	MB 8.1, CV 5	3.778, 1.251, 0.195	360 min, UV light	98 CV and 88.68 MB	1.751–1.801	/	6 runs: 86%	[21]
SnSe nanostructure	facile chemical	MB	/	/	/	20 min, visible light	92.91	/	/	/	[22]
NiSe-NPs	Hydrothermal	MB, 50mL	50mg	/	/	120 min,	92	1.74	0.0188	/	[23]

						visible light			6		
CNF/Ag_2Se films	situ synthesis	MB, 20mg/L	10% Ag_2Se	3-9	/	60 min, visible light	50, 95, 99	4.61-2.71 eV	0.076	Stable and reusable for 5 cycles.	[24]
ZnSe/CoSe Nano composite	Hydrothermal	MB, 20 mg/L	/	/	1.0004	120 min, visible light	99.4	2.63	/	4 runs: 80%	[25]
$PtSe_2$/graphene hybrid materials	Microwave assisted	MB, 0.1 mg mL^{-1}	10 mg /80 mL	/	/	30–180 min, visible light	Maximum	2	0.022	/	[26]
CuSe/$MoSe_2$ 2D/2D	Hydrothermal	100 mL MB solution, 1 × 10^{-5} M	20 mg	/	41.2	Hg lamp, Xe Lamp	98	CuSe 1.47, MoSe 1.68	1.71	/	[27]
$MoSe_2$/ZnSe	Hydrothermal	100 mL MB, 2×10^{-5}	20 mg	/	25.6	180 min, mercury	91.5	/	0.447	/	[28]

		M.				lamp					
Cu_3Se_2/rGO nanocomposites	Co-precipitation	MB	/	/	/	150 min, solar light	97	1.9	/	/	[29]
CuSe/$MoSe_2$ 2D/2D	Hydrothermal	100 mL MB solution, 1 × 10^{-5} M	20 mg	/	41.2	Hg lamp, Xe Lamp	98	CuSe 1.47, MoSe 1.68	1.71	/	[27]
$MoSe_2$/ZnSe	Hydrothermal	100 mL MB, 2×10^{-5} M.	20 mg	/	25.6	180 min, mercury lamp	91.5	/	0.447	/	[28]
Cu_3Se_2/rGO	Co-precipitation	MB	/	/	/	150 min, solar light	97	1.9	/	/	[29]

Table 6 Overview of Methyl orange degradation by metal selenide based photocatalysts

Photocatalyst	Synthesis route	Dyes and concentrations	Catalyst dosage	pH	S_{BET} (m^2 g^{-1})	Irradiation time and source	Removal (%)	Eg (eV)	k_{app} (min-1)	References
PANI–Bi_2Se_3 NFs composites	Solvothermal and in-situ oxidation	MO, RhB, MG	25mg/50 ml,	/	/	MO:150 min, RhB: 120 min, MG:10 min, visible light	MO: 96.31 RhB: 97.7 MG: 97.12	1.35	MG: 0.009 RhB: 0.012 MO: 0.001	[30]
$Zn_{1-x}Ni_xSe$ nanostructures	Hydrothermal	MO: 62 µM/L, CR: 29 µM/L, CY: 27 µM/L	0.1g	/	46.28	180 min, visible light	MO=79.1, CR=91.3, CY=23.2	2.58	/	[31]
ZnSe NPs	Co-precipitation	MO, 100 mL	10 mg	/	/	120 min, visible light	800°C: 80, 200°C: 75	/	200°C:0.01143, 800 °C: 0.01229	[32]
ZnO, ZnO/ZnSe, ZnO/ZnSe/$MoSe_2$.	Hydrothermal	MO, 30 mg/L	/	/	ZnO: 14.303, ZnO/ZnSe:	180 min, visible light	91.5	ZnO=3.18, ZnO/ZnSe=2.	/	[33]

					15.952, ZnO/ZnSe/$MoSe_2$: 9.659			94, ZnO/ZnSe/MoSe$_{2 = 2.91}$		
Se-ZnSNCs	Wet Impregnation	MO, 5 ml (40 μM)	20 mg	/	54-75	160 min, UV light	95.51, 55.09	3.30	0.0084	[34]

Table 7 Overview of Rhodamine B degradation by metal selenide based photocatalysts

Photocatalyst	Synthesis route	Dyes and concentrations	Catalyst Dose	pH	S_{BET} (m^2 g^{-1})	Irradiations time and source	Removal (%)	Eg (eV)	kapp (min^{-1})	References
$TiSe_2$	TOP-free chemical solution	RhB, 10 mg/L	/	/	/	30 min, Xe lamp	96	1.44	2.720	[35]
$ZnSeO_3$	Green synthesis	RhB	10mg	8	58	120 min, visible light	99	2.40	4.916	[36]
CoSe-NPs)	Precipitation	RhB, 20 μg/mL	/	/	/	120 min, UV light	/	1.8	0.022	[37]
$PtSe_2$–graphene/TiO_2	Ultrasonic assisted method	RhB, 80mL	10 mg	/	/	180 min, UV/visible light	70	2.20	0.004	[38]
Fe_3O_4/ZnO/ZnSe	Green synthesis	RhB, 360mL	1.0 mg/mL	/	27.85	80 min, solar light	97.8	2.15	/	[39]
Se-NRs	Convenient	RhB, 10^5 M	3.0 mg	9	/	135 min, UV	85	3.55	/	[40]

	green procedure					light				
Se-NRs	Green synthesis	RhB, 10^{-6} M	5.0 mg	10	/	120 min, UV light	85	4.30.	/	[41]
Bi_2Se_3-Nps	Sonochemical	RhB: 5mg/L	20 mg	/	/	120	93, 98	1.66	/	[42]
(Se-NMs)	Biogenic synthesis	MB: 10mg/L	10 mg and 2 mL H_2O_2	4-11	/	300	Complete Degradation	1.74	0.0048	[43]

Table 8 Overview of degradation of Sun set yellow by metal selenide based photocatalyst.

Photocatalyst	Synthesis route	Dyes and concentration	Catalyst Dose	pH	Irradiations time and source	Removal (%)	Eg (eV)	kapp (min^{-1})	References
CS–ZnSe-Nps	Chemical reduction	Tartrazin, SSY, 30 ppm	0.09 g, 0.01 g	5	180 min, sunlight	Tartrazine: 98<br>SSY: 97	/	0.0013	[44].
Se-NPLs	Green synthesis	SSY, 5 mL/L	0.3 g/L	5.8	Solar Light /UV Light	solar light: 83.8,<br>UV light: 76.6	2.3	0.173	[45].

Reference

1. Azam, J., et al., *Solar Light Assisted Metal Selenide-Chitosan Nanoparticles from Structure Tailoring to Photocatalytic Mineralization of Selected Industrial Dyes.* Water, Air, & Soil Pollution, 2024. **235**(6): p. 386.
2. Ali, N., et al., *Selenide-chitosan as high-performance nanophotocatalyst for accelerated degradation of pollutants.* Chemistry–An Asian Journal, 2020. **15**(17): p. 2660-2673.
3. Khan, S., et al., *Degradation of Congo red dye using ternary metal selenide-chitosan microspheres as robust and reusable catalysts.* Environmental Technology & Innovation, 2021. **22**: p. 101402.
4. Yang, Y., et al., *Chitosan-capped ternary metal selenide nanocatalysts for efficient degradation of Congo red dye in sunlight irradiation.* International Journal of Biological Macromolecules, 2021. **167**: p. 169-181.
5. Yasmin, N., et al., *Synthesis and characterization of silver-indium and antimony selenide: role in photocatalytic degradation of dyes.* Heliyon, 2022. **8**(10).
6. Ali, N., et al., *Regenerable chitosan-bismuth cobalt selenide hybrid microspheres for mitigation of organic pollutants in an aqueous environment.* International Journal of Biological Macromolecules, 2020. **161**: p. 1305-1317.
7. Kulsi, C., et al., *Enhanced photo catalytic performance of nickel doped bismuth selenide under visible light irradiation.* Materials Research Express, 2017. **4**(3): p. 035902.
8. Yasmin, N., et al., *Synthesis, characterization, and photocatalytic activity of quaternary selenide composite using natural sunlight.* Optik, 2022. **251**: p. 168460.
9. Ahmad, W., et al., *Photocatalytic degradation of crystal violet dye under sunlight by chitosan-encapsulated ternary metal selenide microspheres.* Environmental Science and Pollution Research, 2021. **28**(7): p. 8074-8087.
10. Mahmood, A., et al., *Room Temperature Assisted Copper Selenide Based Composite for Outstanding Crystal Violet Degradation.* Available at SSRN 4683682.
11. Kulsi, C., et al., *Remarkable photo-catalytic degradation of malachite green by nickel doped bismuth selenide under visible light irradiation.* Applied Surface Science, 2017. **392**: p. 540-548.
12. Ahmed, M.K., et al., *Silver-doped cadmium selenide/graphene oxide-filled cellulose acetate nanocomposites for photocatalytic degradation of malachite green toward wastewater treatment.* ACS omega, 2021. **6**(36): p. 23129-23138.
13. Abdullah, M., et al., *Visible-light-driven 2D carbonaceous-based SnSe nanohybrid soft materials: A photocatalyst for efficient photo reduction of malachite green dye.* Journal of Molecular Liquids, 2024. **401**: p. 124668.
14. Saied, E., et al., *Aspergillus terreus-mediated selenium nanoparticles and their antimicrobial and photocatalytic activities.* Crystals, 2023. **13**(3): p. 450.
15. Sridevi, D., V. Ramesh, and E. Sundaravadivel, *Ultraviolet light induced dye degradation of methylene blue in the presence of photocatalytic CdSe and ZnSe nanoparticles.* Materials Today: Proceedings, 2021. **42**: p. 1244-1250.

16. Mosavi, S.A., et al., *Photocatalytic removal of Methylene Blue using Ag@ CdSe/Zeoilte nanocomposite under visible light irradiation by Response Surface Methodology.* Materials Chemistry and Physics, 2021. **267**: p. 124696.
17. Hsieh, S., W. Chen, and T. Yeh, *Degradation of methylene blue using ZnSe–graphene nanocomposites under visible-light irradiation.* Ceramics International, 2015. **41**(10): p. 13759-13766.
18. Patel, P., et al., *Photocatalytic degradation of methylene blue dye using copper selenide nanoparticles prepared by co-precipitation method.* Journal of Cluster Science, 2024. **35**(1): p. 191-199.
19. Gharbani, P., A. Mehrizad, and S.A. Mosavi, *Optimization, kinetics and thermodynamics studies for photocatalytic degradation of Methylene Blue using cadmium selenide nanoparticles.* npj Clean Water, 2022. **5**(1): p. 34.
20. Patel, P., et al., *Sunlight-driven catalytic degradation of MB dye and multi-cycle Re-usability analysis of Cu2-xSe nanoparticles.* Heliyon, 2024. **10**(21).
21. Patel, K., et al., *Photocatalytic degradation of methylene blue and crystal violet dyes under UV light irradiation by sonochemically synthesized CuSnSe nanocrystals.* The European Physical Journal Plus, 2021. **136**(7): p. 743.
22. Ashiq, M.N., et al., *Visible-light active tin selenide nanostructures: synthesis, characterization and photocatalytic activity.* New Journal of Chemistry, 2017. **41**(23): p. 14689-14695.
23. Velayutham, L., et al., *Antibacterial and photocatalytic dye degradation activities of green synthesized nise nanoparticles from hibiscus rosa-sinensis leaf extract.* Water, 2023. **15**(7): p. 1380.
24. Hameed, T.A., A. Salama, and R.A. Nasr, *Synthesis of reusable CNF/Ag2Se films as visible-light-driven photocatalysts for photocatalytic degradation of MB.* Journal of Polymers and the Environment, 2024. **32**(9): p. 4440-4455.
25. Yin, H., et al., *Photocatalytic Properties of Zinc Selenide and Cobalt Selenide Nanocomposite.* Journal of the Chemical Society of Pakistan, 2022. **44**(2): p. 89-97.
26. Oh, W.-C., et al., *Photocatalytic properties under visible light with graphene based platinium selenide nanocomposites synthesized by microwave assisted method.* Materials science in semiconductor processing, 2014. **25**: p. 34-42.
27. Cheng, Y., et al., *High photocatalytic and photoelectrochemical performances of the CuSe/MoSe2 2D/2D face-to-face heterojunction photocatalyst.* Journal of Alloys and Compounds, 2021. **870**: p. 159540.
28. Tian, P., et al., *High photocatalytic and photoelectrochemical performance of a novel 0D/2D heterojunction photocatalyst constructed by ZnSe nanoparticles and MoSe2 nanoflowers.* Ceramics International, 2020. **46**(9): p. 13651-13659.
29. Nouri, M., et al., *High solar-light photocatalytic activity of using Cu3Se2/rGO nanocomposites synthesized by a green co-precipitation method.* Solid State Sciences, 2017. **73**: p. 7-12.
30. Chatterjee, M.J., et al., *Visible-light influenced photocatalytic activity of polyaniline-bismuth selenide composites for the degradation of methyl orange, rhodamine B and malachite green dyes.* Applied Surface Science, 2019. **470**: p. 472-483.

31. Karuppasamy, K., et al., *Ternary Zn1-xNixSe nanostructures as efficient photocatalysts for detoxification of hazardous Congo red, methyl orange, and chrome yellow dyes in wastewater sources.* Environmental Research, 2021. **201**: p. 111587.
32. Beena, V., et al., *Enhanced photocatalytic and antibacterial activities of ZnSe nanoparticles.* Journal of Inorganic and Organometallic Polymers and Materials, 2021. **31**(11): p. 4390-4401.
33. Yang, Y., et al., *Insights into the mechanism of enhanced photocatalytic dye degradation and antibacterial activity over ternary ZnO/ZnSe/MoSe2 photocatalysts under visible light irradiation.* Applied Surface Science, 2021. **539**: p. 148220.
34. Ahluwalia, S., et al., *Improved degradation of methyl orange dye using bio-co-catalyst Se nanoparticles impregnated ZnS photocatalyst under UV irradiation.* Chemical engineering journal, 2016. **306**: p. 1041-1048.
35. Ge, W., et al., *Trioctylphosphine (TOP)-free synthesis of TiSe2 plates for enhanced photocatalytic degradation performance of Rhodamine B dyes.* Solid State Sciences, 2020. **103**: p. 106189.
36. Anitha, A., et al., *Novel biosynthesized zinc selenite photocatalysts for enhanced degradation of oxytetracycline and Rhodamine B dye with antibacterial activity.* BioMetals, 2025. **38**(2): p. 505-527.
37. Firdaus, F., M.S. Khan, and U. Baig, *Facile synthesis, characterization and photocatalytic activity of band gap engineered cobalt selenide nanoparticles.* Arabian Journal for Science and Engineering, 2016. **41**(6): p. 2377-2384.
38. Ullah, K., et al., *Optical and photocatalytic properties of novel heterogeneous PtSe2–graphene/TiO2 nanocomposites synthesized via ultrasonic assisted techniques.* Ultrasonics sonochemistry, 2014. **21**(5): p. 1849-1857.
39. Xu, G., et al., *Continuous high-efficient degradation of organic pollutants based on sea urchin-like Fe3O4/ZnO/ZnSe heterostructures in photocatalytic magnetically fixed bed reactor.* Colloids and Surfaces A: Physicochemical and Engineering Aspects, 2020. **603**: p. 125198.
40. Velayati, M., et al., *Biosynthesis of Se-Nanorods using Gum Arabic (GA) and investigation of their photocatalytic and cytotoxicity effects.* Inorganic Chemistry Communications, 2021. **128**: p. 108589.
41. Velayati, M., et al., *Green-based biosynthesis of Se nanorods in chitosan and assessment of their photocatalytic and cytotoxicity effects.* Environmental Technology & Innovation, 2022. **27**: p. 102610.
42. Ramezani, M., A. Sobhani-Nasab, and A. Davoodi, *Bismuth selenide nanoparticles: simple synthesis, characterization, and its light harvesting applications in the presence of novel precursor.* Journal of Materials Science: Materials in Electronics, 2015. **26**(7): p. 5440-5445.
43. Che, L., et al., *Characterization of selenite reduction by Lysinibacillus sp. ZYM-1 and photocatalytic performance of biogenic selenium nanospheres.* ACS Sustainable Chemistry & Engineering, 2017. **5**(3): p. 2535-2543.
44. Zhang, S., et al., *Designing, characterization, and evaluation of chitosan-zinc selenide nanoparticles for visible-light-induced degradation of tartrazine and sunset yellow dyes.* Environmental Research, 2022. **213**: p. 113722.

45. Hassanien, R., A.A. Abed-Elmageed, and D.Z. Husein, *Eco-friendly approach to synthesize selenium nanoparticles: Photocatalytic degradation of sunset yellow azo dye and anticancer activity.* ChemistrySelect, 2019. **4**(31): p. 9018-9026.
